# Characterization of Transcriptional Expression and Regulation of Carotenoid Cleavage Dioxygenase 4b in Grapes

**DOI:** 10.3389/fpls.2020.00483

**Published:** 2020-05-08

**Authors:** Nan Meng, Yi Wei, Yuan Gao, Keji Yu, Jing Cheng, Xiang-Yi Li, Chang-Qing Duan, Qiu-Hong Pan

**Affiliations:** ^1^Center for Viticulture and Enology, College of Food Science and Nutritional Engineering, China Agricultural University, Beijing, China; ^2^Key Laboratory of Viticulture and Enology, Ministry of Agricultural and Rural Affairs, Beijing, China

**Keywords:** carotenoid cleavage dioxygenase 4b, VvMADS4, norisoprenoids, *Vitis vinifera* L., expression and regulation

## Abstract

Norisoprenoids are important aromatic volatiles contributing to the pleasant floral/fruity odor in grapes and wine. They are produced from carotenoids through the cleavage of carotenoid cleavage dioxygenases (CCDs). However, the underlying mechanisms regulating *VvCCD* expression remain poorly understood. In this study, we showed that *VvCCD4b* expression was positively correlated with the accumulation of β-damascenone, β-ionone, 6-methyl-5-hepten-2-one, geranylacetone, dihydroedulan I, and total norisoprenoids in developing grapes in two vintages from two regions. *VvCCD4b* was found to be principally expressed in flowers, mature leaves, and berries. Abscisic acid strongly induced the expression of this gene. Additionally, the present study preliminarily indicated that the activity of the *VvCCD4b* promoter was dropped under 37°C treatment and also responded to the illumination change. *VvCCD4b* was expressed in parallel with *VvMADS4* in developing grape berries. The latter is a MADS family transcription factor and nucleus-localized protein that was captured by yeast one-hybrid. A dual-luciferase reporter assay in tobacco leaves revealed that VvMADS4 downregulated the activity of the *VvCCD4b* promoter. *VvMADS4* overexpression in grape calli and *Vitis quinquangularis* Rehd. leaves repressed the *VvCCD4b* expression. In summary, this work demonstrates that *VvCCD4b* expression is positively correlated with the accumulation of norisoprenoids, and VvMADS4 is a potential negative regulator of *VvCCD4b*. Our results provide a new perspective for understanding the regulation of *VvCCD4b* expression and norisoprenoid accumulation in grapes.

## Introduction

Norisoprenoids are volatile C_9_, C_10_, C_11_, and C_13_ molecules with a low odor perception threshold, generating the floral and fruity scents in grape berries and wine. The content of monoterpenes is extremely low in neutral grape varieties and their corresponding wines (e.g., *Vitis vinifera* L. cv. Cabernet Sauvignon, Merlot, Syrah, and Chardonnay). In contrast, norisoprenoids are more important than other terpenoids in forming the characteristic aromas of these varieties, because the norisoprenoid concentrations tend to be higher than their thresholds (Mateo and Jiménez, 2000). Indeed, several norisoprenoids are the primary contributors to aroma in red and white grapes and wines (Xu et al., 2015; Gao et al., 2016; Asproudi et al., 2018; Chen W. K. et al., 2018). For example, β-damascenone exhibits a complex smell of “cooked apple,” “floral,” and “quince” (Kotseridis et al., 1999), whereas β-ionone was described as “violet,” “woody,” and “raspberry” (Aznar et al., 2001). Aged Riesling wines have a floral profile of “kerosene” and “petrol” owing to the presence of 1,1,5-trimethyl-1,2-dihydronaphthalene (TDN) (Simpson, 1978; Simpson and Miller, 1983).

Carotenoids are the precursors of norisoprenoids, and they are synthesized via the methyl-erythritol-phosphate (MEP) pathway in plastids (Dunlevy and Kalua, 2009). Major enzymes driving biochemical reactions in this pathway include deoxyxylulose 5-phosphate synthase (DXS), deoxyxylulose 5-phosphate reductoisomerase (DXR), and 1-hydroxy-2-methyl-2-(*E*)-butenyl-4-PP reductase (HDR) (Cunningham and Gantt, 2000; Dunlevy and Kalua, 2009). Besides, carotenoid cleavage dioxygenases (CCDs) are critical enzymes that catalyze the generation of norisoprenoids (apocarotenoids) by cleaving the conjugate double bond of carotenoids (Fleischmann et al., 2003; Castillo et al., 2005; Baldermann et al., 2010; Campbell et al., 2010; Chiou et al., 2010; Ledger et al., 2010; Brandi et al., 2011; Frusciante et al., 2014). In *Arabidopsis thaliana*, AtCCD1 and AtCCD4 cleave carotenoids to produce norisoprenoid compounds. The CCD1 and CCD4 homologs from some other plants were also reported to possess these biochemical functions, although they show different preferences toward the substrates and the cleavage sites (Fleischmann et al., 2003; Castillo et al., 2005; Baldermann et al., 2010; Campbell et al., 2010; Chiou et al., 2010; Ledger et al., 2010; Brandi et al., 2011; Adami et al., 2013; Ma et al., 2013; Frusciante et al., 2014; Bai et al., 2015). CCD4 usually has more than one isoform in plants, involving the coloration of petals or the generation of floral/fruity aroma by cleaving carotenoids. In *Chrysanthemum morifolium*, CmCCD4a, which mostly expressed in petals, cleaves the carotenoid, leading to the formation of a white flower (Ohmiya et al., 2006). CsCCD4c cleaves β-carotene at different sites to produce β-ionone and β-cyclocitral; meanwhile, this enzyme also uses lutein, neoxanthin, and violaxanthin as substrates (Rubio-Moraga et al., 2014). In grapevines, VvCCD1, VvCCD4a, and VvCCD4b have been biochemically characterized. VvCCD1 isolated from “Shiraz” can cleave zeaxanthin to generate 3-hydroxy-β-ionone (Mathieu et al., 2005). Overexpressing *VvCCD1*, *VvCCD4a*, and *VvCCD4b* from *V. vinifera* L. cv “Pinotage” in carotenoid-generating *Escherichia coli* strains revealed that they all can cleave lycopene and ε-carotene to form 6-methyl-5-hepten-2-one (MHO) and β-ionone, respectively (Lashbrooke et al., 2013). VvCCD1 was the only enzyme capable of cleaving β-carotene and incapable of cleaving neurosporene. In another experiment, *VvCCD1* and *VvCCD4b* from “Cabernet Sauvignon” were expressed in recombinant *Saccharomyces cerevisiae* cells that were able to produce carotenoids; both enzymes were found to cleave lycopene and β-carotene (Meng et al., 2019). The three genes are expressed lowly in young grape berries but increased with ripening (Lashbrooke et al., 2013).

Individual norisoprenoid has a distinct accumulation pattern. In “Pinot noir” grapes, total β-damascenone, vitispirane, and TDN increased during ripening, whereas total α-ionone and β-ionone decreased (Yuan and Qian, 2016). In “Cabernet Sauvignon” grapes, the contents of MHO and geranylacetone were nearly identical between the two producing regions in some stages of development (Chen et al., 2017). Actually, environmental factors markedly influence norisoprenoid production in grape berries. Light exposure and water deficit increase the concentration of norisoprenoids (Bindon et al., 2007; Kwasniewski et al., 2010; Feng et al., 2015). The three *VvCCD* genes present some differences in the spatial and temporal expression patterns. *VvCCD4b* is expressed predominantly in mature grape berries (Lashbrooke et al., 2013). Our previous research also observed that norisoprenoid concentration was correlated with the transcript level of *VvCCD4b* in developing “Cabernet Sauvignon” grape berries (Chen et al., 2017). However, the regulation of *VvCCD4b* expression is poorly understood.

In plants, *cis*- and *trans*-acting elements are largely responsible for regulating gene transcription. *Cis*-acting elements in the promoter region determine temporal and spatial gene expression patterns, as well as the manner in which these patterns respond to stress. CmCCD4a-5 in *C. morifolium* and *AtCCD7* in *A. thaliana* are petal specific (Imai et al., 2013) and vascular tissue specific (Liang et al., 2011), respectively. In *Malus*, drought, waterlogging, and methyl jasmonate treatments all decreased *CCD7* promoter activity (Yue et al., 2015).

*Trans*-acting elements, especially transcription factors, bind to the *cis*-acting regulatory regions and influence gene expression. Major transcription factor families in plants include WRKY, MYB, NAC, bZIP, and MADS, and they participate in numerous functions such as stress response, metabolism, and hormonal induction. However, we know little about transcription factors that regulate *CCD* expression. A recent study in *Citrus sinensis* found that CsMADS6 activated *CsCCD1* expression (Lu et al., 2018). Additionally, OfWRKY3 and OfERF61 in *Osmanthus fragrans* Lour. stimulate *OfCCD4* expression, which results in carotenoid cleavage and influences β-ionone synthesis in sweet osmanthus petals (Han et al., 2016, 2019). However, until now, there has been no report involving the transcriptional regulation of *VvCCD*s in grapes.

Thus, in this study, we examined the relationship between *CCD* expression and norisoprenoid accumulation in “Cabernet Sauvignon” grapes of two vintages from two regions with very different climates. We also assessed the temporal and spatial expression patterns of *VvCCD4b* and the responses of its promoter to temperature, light, and abscisic acid (ABA) treatments. Furthermore, we identified a transcription factor potentially regulating the *VvCCD4b* expression. The findings help clarify the mechanisms underlying norisoprenoid biosynthesis in grape berries.

## Materials and Methods

### Plant Materials

This study used “Cabernet Sauvignon” in 2013 and 2016 vintages from the Changli (CL) and Gaotai (GT) regions. These two regions are located in Hebei Province of Northeastern China and Gansu Province of Northwestern China, respectively, and are important wine-producing zones with a monsoon climate and continental climate, respectively. Details on regional climate, vineyards, grapevine cultivation, and sampling method are described in our previous publications (Xu et al., 2015; Chen et al., 2017). Grapes were sampled according to the E-L system (Coombe, 1995). In 2013, berries were sampled 4 (E-L31), 6 (E-L34), 8 (E-L35), 10 (E-L36), 12 (E-L37), and 14 (E-L38) weeks after flowering (WAF) in GT and 6 (E-L31), 8 (E-L34), 10 (E-L35), 12 (E-L36), 14 (E-L37), and 16 (E-L38) WAF in CL. In 2016, GT samples were collected 4 (E-L31), 7 (E-L35), 14 (E-L37), and 16 (E-L38) WAF, and CL samples were collected 6 (E-L31), 9 (E-L35), 16 (E-L37), and 20 (E-L38) WAF. Three biological replicates were performed per sampling. Each replicate randomly collected 300 berries without physical damage or disease, from 150 clusters (Xu et al., 2015). Grape berries with 2-mm pedicels were placed into a plastic bag and then a foam box and transported into the nearest laboratory within 2 h (Wen et al., 2015). At the laboratory, they were frozen in liquid nitrogen and stored at −80°C.

For spatial and temporal expression analyzes, stems, flowers, young leaves (10 days of age), mature leaves (1 month of age), tendrils, and roots were sampled at the full-bloom stage, and grape berries were gathered at 5, 15, 63, 70, 80, and 98 days after flowering following the E-L system from E-L29 to E-L38 (Coombe, 1995) in 2019. All the samples were collected from the self-rooted “Cabernet Sauvignon” grapevines at the Chateau SunGod Great Wall (Huailai, Hebei Province). Except for roots, other tissues were sampled from nine grapevines, which were divided into three groups (three grapevines each group) corresponding to three biological replicates. As for individual tissue, material from one biological replicate was pooled and frozen in liquid nitrogen. They were powdered prior to use. The roots were dug out from the underground parts of another nine grapevines in the same vineyard, which were also divided into three biological replicates. The land is privately owned, and no protected species were sampled (manager: Qing-quan Yu, e-mail: yuqq@cofco.com).

The “Cabernet Sauvignon” grape calli were induced from pulp cells. Callus incubation followed the method described by Wang H. et al. (2015). Wild-type calli were cultured on B5 plates (3.21 g/L B5 basic medium, 30 g/L sucrose, 2.5 g/L acid-hydrolyzed casein, 0.2 mg/L KT, 0.1 mg/L NAA, and 3.0 g/L plant gel; pH 5.9–6.0) at 25°C in the dark. Transgenic calli were cultured on B5 plates with 5 mg/L hygromycin. Both kinds of calli were subcultured every 20–25 days.

Wild-type *A. thaliana* (ecotype Columbia) and tobacco (*Nicotiana benthamiana*) were grown in soil in a greenhouse under a 16 h/8 h light/dark cycle at 23°C.

### Detection of Norisoprenoid Volatiles

Sample pretreatment was performed according to the published method (Lan et al., 2016), with some modifications. Briefly, pedicels and seeds were removed from 100 g grape berries; the remainder was powdered and then blended with 1 g polyvinylpolypyrrolidone and 0.5 g D-gluconic acid lactone under liquid nitrogen. After being macerated at 4°C for 4 h, the clear juice was collected through centrifugation at 6,000 × *g* for 10 min at 4°C. The juice of 5 ml, added with 1 g NaCl and 10 μl internal standard (4-methyl-2-pentanol), was used to detect the free-form volatile compounds using a headspace solid-phase microextraction (HS-SPME) gas chromatography–mass spectrometer (GC-MS). Another 4 ml juice was used to extract glycosidically bound volatile compounds using Cleanert PEP-SPE resin. The extract was dissolved in 10 ml citric acid/sodium citrate buffer (0.2 M, pH 2.5), moved averagely into two 20 ml vials containing 1 g NaCl, and then acid-hydrolyzed to release volatile aglycone (Feng et al., 2015). The vials were tightly capped and incubated for 1 h in a 99°C water bath. After cooling to room temperature, 10 μl of internal standard (4-methyl-2-pentanol) was added to each vial for the detection of volatile aglycones using HS-SPME GC-MS. Two technical replicates of the free-form or bound-form detection were performed per biological replicate. Norisoprenoid compounds were identified and quantified according to the published methods (Xu et al., 2015). The standards β-damascenone, β-ionone, geranylacetone, and MHO were used to establish standard curves in this research. The total concentration (free form + bound form) of each compound was used for further analysis in this study. And the norisoprenoid concentration was expressed as micrograms per berry. β-Damascenone-*Z* and β-damascenone-*E* were individually identified and quantified, and their concentrations, named β-damascenone, were added together for *k*-means analysis and correlation analysis.

### Total RNA Extraction, Reverse Transcription, Quantitative Real-Time PCR, and Reverse Transcription PCR

RNA was extracted from different samples using different amounts. For a single RNA extraction, at least 10 frozen grape berries, half a leaf, or two clumps of 1 cm diameter calli were needed. At least 0.3 g of other samples (stems, roots, flowers, etc.) was needed once. All the frozen samples were ground in liquid nitrogen. Total RNA extraction of grape samples used the Spectrum^TM^ Plant Total RNA Kit (Sigma-Aldrich, St. Louis, MO, United States), and the other materials used E.Z.N.A.^®^ Plant RNA Kit (Omega, Norcross, GA, United States). Both extraction processes used on-column DNase I (Promega, Durham, United Kingdom). The quality and concentration of the obtained RNA were detected by agarose gel electrophoresis using a NanoDrop 2000 spectrophotometer (Thermo Fisher Scientific, MA, United States). RNA for further analysis required showing clear and bright bands in the agarose gel; the value of OD_260_/OD_230_ was more than 1.8, and the value of OD_260_/OD_280_ was between 1.8 and 2.1. First-strand cDNA was synthesized from 1 μg total RNA in a 20 μl reaction mixture following the protocol of HiScript^®^ II Q RT SuperMix for qPCR + gDNA wiper (Vazyme, Nanjing, China).

Quantitative real-time PCR (qRT-PCR) was performed with 2 μl of cDNA as the template using ChamQ Universal SYBR qPCR Master Mix (Vazyme); the *Ubiquitin* gene was used as the reference. The number of PCRs per gene comprised at least three biological replicates and three technical runs of each replicate (at least nine values). The biological replicate number was noted in the experimental methods. Thermocycling conditions (Wen et al., 2015) and analysis methods (Ruijter et al., 2009) were described previously. The dissolution curve illustrated the specificity of the primers. The size of the amplicon was examined by agarose gel electrophoresis, and its nucleotide sequence was confirmed by sequencing.

Reverse-transcription PCR (RT-PCR) was performed, referencing the published methods with some modification (Chang-ho et al., 2015; Tanabe et al., 2015). 2 × Taq PCR MasterMix (KT201) (Tiangen Biotech, Beijing, China) was used for PCR in which a 25 μl reaction mixture contained 1 μl of cDNA template, 2 μl RT-PCR primers, and 9.5 μl ddH_2_O. The internal control was *AtActin8* (Tanabe et al., 2015). DNA polymerase was first activated at 94°C for 5 min, and PCR was run for 25 cycles of 30 s at 94°C, 30 s at 55°C, and 1 min at 72°C, followed by a final extension step for 5 min at 72°C. Products were visualized through agarose gel electrophoresis. And ImageJ software (NIH, Bethesda, United States) was used to measure the bright intensity of bands on an agarose gel. The relative brightness of the target gene was normalized by that of *AtActin8*.

The primer sequences, primer concentrations, and length of the amplicons are listed in Supplementary Table S1.

### Cloning, Sequence Analysis, and Activity Assay of the V*vCCD4b* Promoter

The region ∼1.0 kb at the upstream of start codon ATG was regarded as the *VvCCD4b* promoter (*P_CCD__4__b_*), based on the genomic sequence of “Pinotage^1^” Genomic DNA from “Cabernet Sauvignon” grape berries was obtained using a plant genome extraction kit (Bioteke, Beijing, China). The fragment of *P_CCD__4__b_* was amplified using a gene-specific primer pair, promoter-F/R, designed by Primer Premier 5.0 (Premier Biosoft, United States), and then inserted into a T-vector (Tsingke, Beijing, China) for sequencing. The *cis*-acting elements on *P_CCD__4__b_* were predicted using PLACE^2^ and PlantCARE^3^. The transcription initiation site was identified using TSSP^4^.

To verify the promoter activity, we introduced *P_CCD__4__b_* into a modified pCAMBIA 1300-LUC vector carrying a luciferin (*LUC*) reporter gene (Shang et al., 2010) using homologous recombination (Clontech, Mountain View, CA, United States) with the *Kpn*I and *Sma*I restriction sites. The recombinant vector was transferred into *Agrobacterium tumefaciens* strain GV3101 using the freeze–thaw method (Hofgen and Willmitzer, 1988). Detailed methods on tobacco (*N. benthamiana*) infection and LUC detection were described by Sun et al. (2015).

### Construction of Transgenic *A. thaliana*

A pCAMBIA 1381-P*_CCD__4__b_*-β-glucuronidase (GUS) plasmid was constructed by inserting *P_CCD__4__b_* using homologs recombination with *Sma*I and *Sal*I into pCAMBIA 1381 (CambiaLabs) carrying the *GUS* gene. The construct was transferred into GV3101 and then transformed into *A. thaliana* using the floral-dip method (Clough and Bent, 1998). Transgenic *Arabidopsis* plants were selected from Murashige–Skoog (MS) medium plates (4.43 g/L MS, 30 g/L sucrose, and 8 g/L agar; pH 5.9–6.0) with 50 mg/L hygromycin, cultured in the greenhouse with a 16-h/8-h light/dark cycle at 23°C for 2 weeks. Then the healthy seedlings were transplanted into soil, also cultured in the same greenhouse.

### Abiotic Treatments of Transgenic *A. thaliana*, Grapevine Calli, and Grape Berries

Four-week-old T3-generation transgenic *A. thaliana* individuals (from Construction of Transgenic *A. thaliana*) were divided into four groups and placed in climate chambers for 16 h under the following conditions: 25°C (control), 37°C, 30°C, or 10°C. A different line of transgenic 4-week-old seedlings was subjected to illumination treatments [4,240 (control), 6,630, 1,315, and 0 lx] in a climate chamber for 16 h. Three biological replicates were maintained. Leaves were sampled immediately after the treatments to detect *GUS* expression through RT-PCR. Histochemical staining was also performed to determine GUS activity in plants as described previously (Cho and Cosgrove, 2000).

“Cabernet Sauvignon” grape calli were grown on B5 medium plates containing different ABA concentrations (0, 0.4, 0.8, 1.0, and 2.0 mg/L) for 25 days and then sampled for RNA extraction. Five clumps of callus were placed on one plate, and one concentration treatment used three plates, serving as three biological replicates.

ABA spraying treatment was also performed on Chateau SunGod Great Wall plants in 2019. The three center rows of a “Cabernet Sauvignon” vineyard, containing an average of 30 plants per row in a north–south orientation, were selected. Six vines were chosen randomly and divided into two groups in each row. One group was sprayed with 1 g/L ABA and 0.05% Tween 20 when 5% of the berries reached veraison (began coloration). Another group, as the control, was sprayed with 0.05% Tween 20 only. The sampling was carried out every 2 days until 10 days post-spraying. Approximately 100 berries were collected for each biological replicate per sampling and used for RNA extraction.

### Yeast One-Hybrid Assay

A yeast one-hybrid (Y1H) assay was performed by using the Matchmaker Gold Yeast One-Hybrid Library Screening System (Clontech). The short fragment of the *VvCCD4b* promoter (205–893 bp upstream of ATG of *VvCCD4b*) was inserted into pAbAi to construct the bait vector pAbAi-P_VvCCD__4__b_. The bait vector was linearized and transformed into *S. cerevisiae* Y1HGold to create bait strain. A cDNA library of “Cabernet Sauvignon” was constructed by Clontech. The cDNA library was transformed into the bait strain and screened using synthetic dropout medium (SD) lacking leucine (–Leu) with aureobasidin A (AbA). The prey fragments from the positive colonies were identified by DNA sequencing (Sangon) using the primer pair of pGADT7-F/R and blasted by NCBI.

### Dual-Luciferase Activity Assay in a Transient Expression System

Dual-luciferase activity was assayed in a tobacco transient expression system. *P*_CCD4b_ was subcloned into a pGreen II 0800 double-reporter vector, and the full-length coding sequence (CDS) of transcription factor genes were subcloned into a pCAMBIA 1301 vector as effectors. Recombinant reporter vector and individual effector vector were transferred into A. *tumefaciens* EHA105. The two A. *tumefaciens* strains were mixed at a 1:1 (v:v) ratio and injected into tobacco leaves (Voinnet et al., 2003). Dual-luciferase activity was measured using a Dual-Luciferase^®^ Reporter Assay System (Promega). Six biological replicates were used in one independent experiment. An independent experiment of each transcription factor was repeated for two to four times.

### Subcellular Localization of V*vMADS4*

The CDS of *VvMADS4* (NM_001281185.1) without the stop codon was amplified, sequenced, and inserted into pEZS-NL to express a green fluorescent protein (GFP) fusion protein. The recombinant plasmid was transformed into onion epidermal cells using a gene gun (Bio-Rad, Hercules, CA, United States). Cells were incubated in the dark at 25°C for 16 h and then co-incubated with 50 mg/L 4’,6-diamidino-2-phenylindole (DAPI) for 20 min as a nucleus localization marker. The samples stained with DAPI were observed under a confocal microscope (Nikon A1, Tokyo, Japan). An empty vector was not used as a control because it could not properly express *GFP*^5^.

### V*vMADS4* Overexpression in Grape Calli and Leaves

To verify the role of VvMADS4 in controlling *VvCCD4b* expression, we overexpressed *VvMADS4* in grapevine calli. After inserting *VvMADS4* CDS into pCXSN, the recombinant plasmid was transformed into GV3101. Calli transformation was performed using an *A. tumefaciens*-mediated method (Van Eck et al., 2006) with some modifications. Each callus was submerged in a medium containing *A. tumefaciens* strains and gently shaken for 6 min. After the medium was dried off, the calli were cultivated on a plate containing a sterile filter paper and T1 medium (B5 medium with 100 μmol/L acetosyringone and 100 mg/L DL-dithiothreitol; pH 5.9–6.0). Cultivation occurred in the dark for 3 days. The calli were subsequently washed using sterile water and sterile water with 400 mg/L cefalexin and 400 mg/L carboxymycin in turn for three times. The calli were then dried and cultivated in the dark on a plate containing T2 medium (B5 medium with 100 mg/L DL-dithiothreitol, 400 mg/L cefalexin, 400 mg/L carboxymycin, and 5 mg/L hygromycin). Concentrations of cefalexin and carboxymycin in the solid medium were gradually decreased until new calli appeared. Transgenic calli were identified by hygromycin gene detection and target gene expression quantitation.

Transient *VvMADS4* overexpression using *Vitis quinquangularis* Rehd leaves was also performed to further verify the function of VvMADS4 on the regulation of *VvCCD4b* expression. *V. quinquangularis* Rehd leaves contain little wax and are easily infiltrated by bacterial solution. The expression construct pCAMBIA 1301-VvMADS4 was transferred into GV3101, and empty pCAMBIA 1301 was used as the control. The transformation method followed the procedure described by Xu et al. (2010), with some modifications. A single colony of the GV3101 strain containing the plasmid was inoculated into 5 ml LB liquid medium (50 mg/L rifampicin and 50 mg/L kanamycin) and cultured at 28°C and 220 rpm for 20 h. Then, 300 μl bacterial liquid was added to 30 ml LB liquid medium (50 mg/L rifampicin and 50 mg/L kanamycin) and cultured at 28°C and 220 rpm until OD_600_ reached 0.5–0.6. Subsequently, the culture was centrifuged at 4°C and 4,000 × *g* for 10 min and then resuspended with induction buffer (2.132 g/L MES, 2.033 g/L MgCl_2_⋅6H_2_O, 5 g/L sucrose, and 0.039 g/L acetosyringone; pH 5.9–6.0) until OD_600_ reached 0.3–0.4. After the bacterial suspension was inoculated at 25°C for 3 h, the leaves were submerged into the suspension and subjected to vacuum (−0.8 MPa) for 20 min to induce infiltration. After 3 days of incubation at 25°C in the dark, the leaves were used for gene expression analysis. This experiment was repeated twice, each time with three biological replicates at least and one leaf per replicate.

### Statistical Analysis

Data are presented as means ± SD (standard deviation). Pearson’s correlation and one-way analysis of variance (ANOVA) were conducted using SPSS for Windows version 20.0 (SPSS Inc., United States). String diagrams, bar charts, and box plots were created in OriginPro 9.0 (OriginLab, Northampton, MA, United States). *k*-means and heatmaps were generated using *k*-mean in the “base” and “pheatmap” packages in R software. Pearson’s correlation analysis of *VvCCD4b* expression and norisoprenoid accumulation was performed after data normalization. The concentration of individual norisoprenoid compound was normalized by dividing the concentration by the maximum value of all samples (over 2 years from both regions) to enable the compounds to be compared, regardless of a wide range of concentrations. The relative expression of *VvCCD*s was also normalized using the same method. Pearson’s correlation analysis was performed using the normalized relative expression of *VvCCD*s and the normalized concentration of norisoprenoids during the entire development stage over 2 years from both regions (Xu et al., 2015). Pearson’s correlation was also estimated between the expression of *VvCCD4b* and candidate transcription factor genes identified by Y1H. The gene expression data were from the RNA-seq dataset^6^. The transcript abundance of each gene was calculated using the FPKM (fragments per kilobase per million fragments mapped) method. The information about the experiment protocol, data analysis of RNA-seq, and submission to the NCBI Gene Expression Omnibus was recorded in the publication of our research group (Sun et al., 2019). In this study, we selected the data of the control group at the E-L29, E-L31, E-L35, E-L36, E-L37, and E-L38 stages of berry development for Pearson’s correlation analysis.

## Results

### Correlation Between V*vCCDs* Expression and Norisoprenoid Content

**FIGURE 1:**
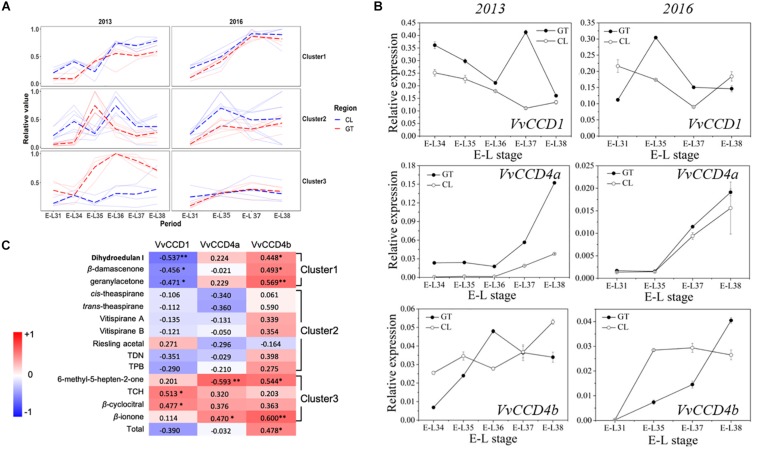
The concentrations of norisoprenoids and the expression levels of *VvCCD*s in grape berries from two regions in 2 years. **(A)** The analysis of *k*-means clustering for 2013 and 2016 vintages. **(B)** The expression of *VvCCDs* in “Cabernet Sauvignon” grape berries from two regions in 2 years. The data are expressed as means ± SD of three replications. **(C)** Heatmap of Pearson’s correlation results. The correlation is assessed based on the normalized concentration of individual norisoprenoid volatile and the normalized expression level of *VvCCDs* during the development period. The data on the heatmap are correlation coefficients. ^∗∗^Significant correlation at *P* < 0.01 (two-sided test). ^∗^Significant correlation at *P* < 0.05 (two-sided test). The red block indicates positive correlation, and the blue block indicates negative correlation. The clusters were corresponding to the results of the *k*-means analysis. TDN: 1,1,5–trimethyl–1,2–dihydronaphthalene; TCH: 2,2,6-trimethylcyclohexanone; TPB: (*E*)-1-(2,3,6-trimethylphenyl) buta-1,3-diene.

We quantified 15 free-form and 6 glycosidically bound-form norisoprenoid compounds (Supplementary Table S2). The total concentration (free form plus bound form) of individual norisoprenoid compound is shown in Supplementary Table S3. The *k*-means analysis divided the norisoprenoids into three clusters based on the total concentration changes during berry development across 2 years (for individual compounds per cluster, see Figure 1C). The compounds in the first cluster accumulated continuously as the grapes matured. The compound concentration in the second cluster peaked between E-L35 and E-L36 before decreasing gradually. Finally, the third cluster maintained a steady concentration, except for the GT grapes in 2013 (Figure 1A). Correspondingly, the *VvCCD* expression patterns differed across development (Figure 1B). *VvCCD*1 expression was higher during early development than during maturation. *VvCCD4a* expression was very low before maturation but increased sharply from E-L36 to E-L37 until ripening. *VvCCD4b* transcription level in ripening grapes was higher than that in green berries. Pearson’s correlation analysis revealed that the accumulation of 2,2,6-trimethylcyclohexanone (TCH) and β-cyclocitral was positively correlated only with *VvCCD1* expression. The accumulation of dihydroedulan I, geranylacetone, and β-damascenone, in cluster 1, highly paralleled with *VvCCD4b* expression but opposed to *VvCCD1* expression. MHO and β-ionone, in cluster 3, were positively correlated with *VvCCD4a* and *VvCCD4b* expression (Figure 1C). Notably, *VvCCD4b* expression was significantly correlated with the total concentration of norisoprenoids, indicating that VvCCD4b is a key enzyme in norisoprenoid production during grape berry development.

### Temporal and Spatial V*vCCD4b* Expression Patterns

**FIGURE 2:**
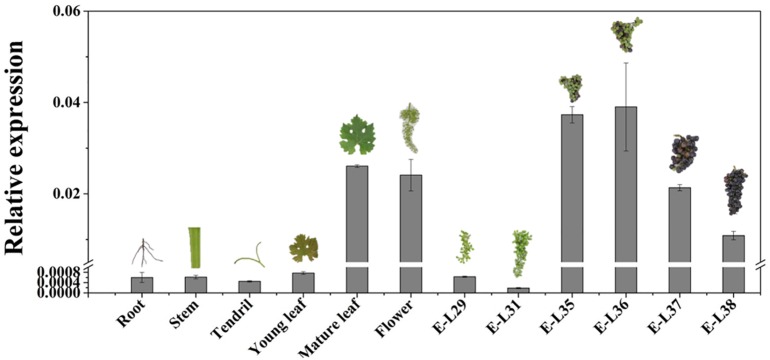
Temporal and spatial expression of *VvCCD4b* in various organs of “Cabernet Sauvignon” grapevine. The data are expressed as means ± SD of three replications.

The temporal and spatial expression pattern of *VvCCD4b* was examined by qRT-PCR. *VvCCD4b* showed low expression levels in the root, stem, and tendril and high expression levels in the flower, mature leaf, and mature fruit (Figure 2). The expression of *VvCCD4b* increased with the age of leaves and the maturation of grape berries. Combining the results of Figures 1B, 2, we found that there is an upregulation of *VvCCD4b* expression at the E-L35 stage.

### Response of V*vCCD4b* Promoter to Light and Temperature

**FIGURE 3:**
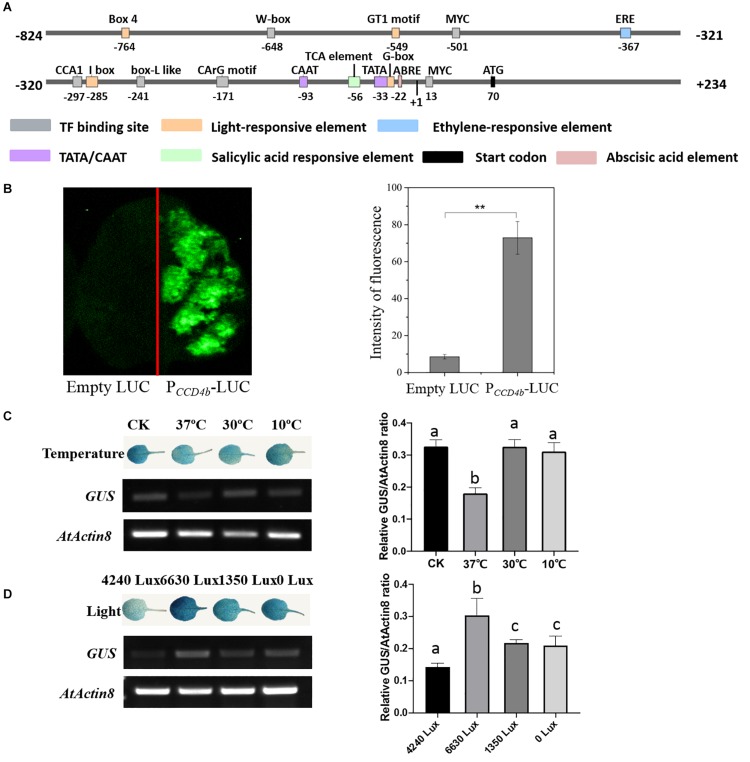
Analysis of *cis*-acting elements and examination of *VvCCD4b* promoter activity. **(A)** The map of the *VvCCD4b* promoter region deduced by the PlantCARE and PLACE programs. **(B)** Transient expression of the *LUC* gene driven by the *VvCCD4b* promoter. Tobacco leaves were transformed with equal amounts of empty LUC plasmid (left side) and P*_CCD__4__b_*-LUC (right side). Left panel: fluorescence imaging. Right panel: intensity of fluorescence analyzed by ImageJ. Asterisks indicate significant differences relative to the control by one-way ANOVA test (**P < 0.01). **(C,D)** Effects of temperatures or illuminations on the expression of the *GUS* gene driven by the *VvCCD4b* promoter in the transgenic *Arabidopsis* leaves. *AtActin8* was used as a control for the RT-PCR. Amplified products were electrophoresed on 1.5% (w/v) agarose gels. The relative *GUS*/*AtActin8* ratio is quantified by ImageJ software according to the brightness of *GUS* and *AtActin8* bands in agarose gels. Data are expressed as means ± SD of three biological replicates. Lowercase letters indicate significant differences analyzed by one-way ANOVA tests. The original figures of the agarose gels in **(C,D)** were shown in Supplementary Figures S2A**,B**, respectively. GUS staining patterns and agarose gel results of two additional biological replicates are shown in Supplementary Figures S2C,D, respectively.

To research the *cis*-acting elements on the *VvCCD4b* promoter and its responses to stresses, we cloned a 1,057-bp *VvCCD4b* promoter from the DNA of “Cabernet Sauvignon.” The promoter shared 99% similarity with the sequence from “Pinot noir” (Supplementary Figure S1). The transcription initiation site, located 70 bp upstream of the start codon (ATG), was set as position + 1 (Figure 3A). A putative TATA-box (TCATTATAAAA) and a CAAT-box (CAAAT) that are necessary for transcription were found at positions −23 to −33 and −93 to −97, respectively. We also found several putative environmental stress-responsive or hormone-responsive *cis*-acting regulatory elements in the *VvCCD4b* promoter. Box 4, GT1 motif, I-box, and G-box are involved in light responsiveness. Ethylene-responsive element (ERE), TCA element, and ABA-responsive element (ABRE) are associated with responses to ethylene, salicylic acid, and ABA, respectively. W-box, MYC binding site, CCA1 binding site, box L-like, and CArG motif are all important binding sites for transcription factors (Supplementary Table S4).

Then, P*_CCD__4__b_*-LUC was transformed into tobacco leaves to identify the activity of the *VvCCD4b* promoter. The transformation of tobacco leaves with a construct containing *VvCCD4b*-promoter-driven *LUC* yielded strong fluorescence (Figure 3B), whereas fluorescence was absent under control conditions (empty vector). These findings indicate that the *VvCCD4b* promoter has activity to drive *LUC* expression.

To test the responses of the *VvCCD4b* promoter to temperature and light treatment, we subcloned *P_CCD__4__b_* into the pCAMBIA 1381 vector to drive the *GUS* gene and then transformed it into *A. thaliana.* The expression levels of *GUS* and *AtActin8* in transgenic *Arabidopsis* were assessed by RT-PCR, and the relative *GUS/AtActin8* ratio was analyzed using the brightness of bands on the agarose gel. The result revealed that *GUS* transcript abundance was lower at 37°C than that at 25°C (the control group), whereas the treatments at 10 and 30°C had no significant effect on the *GUS* expression (Figure 3C). This indicated that the activity of the *VvCCD4b* promoter was repressed by extremely high temperatures. Additionally, strong illumination, weak illumination, and dark treatment all promoted *GUS* transcript accumulation, especially strong light stimulation (Figure 3D). The investigation illustrated that the *VvCCD4b* promoter could respond to illumination changes.

### V*vCCD4b* Response to ABA Treatments in Calli and Grapes

**FIGURE 4:**
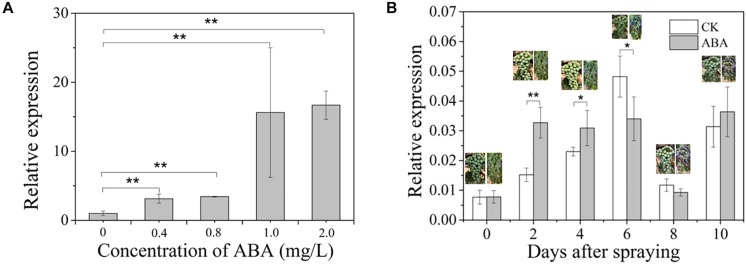
Effects of exogenous ABA treatment on *VvCCD4b* expression. **(A)** The relative expression of *VvCCD4b* in calli treated by ABA with different concentrations. **(B)** The relative expression of *VvCCD4b* in grape berries after being sprayed by ABA or Tween 20 solution. The data are expressed as means ± SD of three replications in both figures. Asterisks indicate significant differences relative to the control by one-way ANOVA test (*P < 0.05; **P < 0.01).

Given that an upregulation of *VvCCD4b* expression was observed at E-L35 (the onset of berry ripening) (Figure 1B) and that an ABRE element was present in the *VvCCD4b* promoter, we hypothesized that the grape ripening hormone ABA may be involved in the induction of *VvCCD4b* expression. To test this hypothesis, we treated the grape calli with ABA of different concentrations for 25 days. The results verified that ABA upregulated *VvCCD4b* expression, with the latter increasing with increasing ABA (Figure 4A).

Afterward, we sprayed ABA solution on grape berries at the onset of ripening (5% berry coloration) to test if ABA had a similar effect on *VvCCD4b* expression in developing grape berries. The result showed that immediately after ABA spraying, the coloration of berries was accelerated and that *VvCCD4b* expression was upregulated compared to that in the control; however, this effect disappeared gradually. *VvCCD4b* expression amount in the treated group was about 2-fold and 1.34-fold greater than that in the control at 2 and 4 days after ABA spraying, respectively (Figure 4B). On Day 6 post-spraying, the expression level of *VvCCD4b* in the treated group was lower than that in the control. Subsequently, *VvCCD4b* transcript abundance ceased to differ between the ABA-treated group and the control. Both experiments of grape calli and berries suggested that *VvCCD4b* expression is induced by ABA, explaining the upregulation of *VvCCD4b* expression at E-L35.

### Identification of Transcription Factors Regulating V*vCCD4b* Expression

**FIGURE 5:**
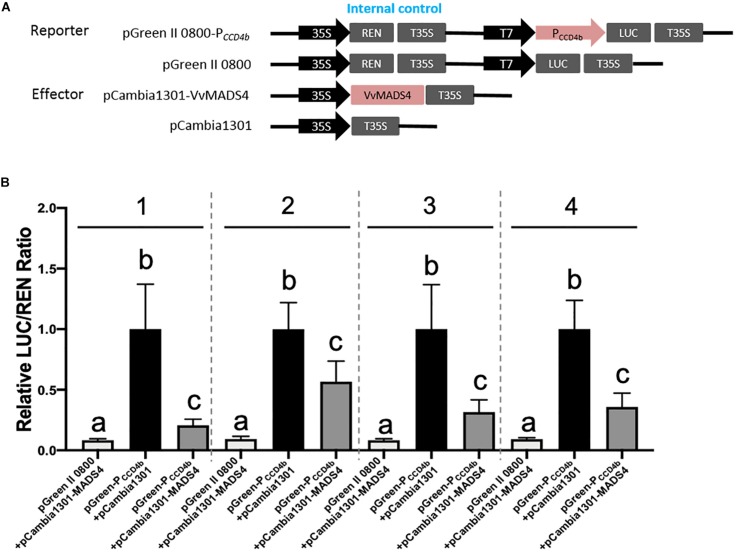
Regulation of VvMADS4 on the activity of the *VvCCD4b* promoter. **(A)** Schematic diagrams of vectors used for the dual-luciferase assay. The pGreen II 0800, empty, pGreen-P*_CCD__4__b_*, and reporter vectors contained the *VvCCD4b* promoter fused to LUC. The pCAMBIA 1301, empty, pCAMBIA 1301-VvMADS4, and overexpression vectors contained VvMADS4. **(B)** The dual-luciferase assay was performed using a tobacco transient expression system. pGreen II 0800 + pCAMBIA 1301-VvMADS4 and pGreen-P_CCD__4__b_ + pCAMBIA 1301 are controls. Four independent experiments were performed, and the numbers above the bar chart indicate the number of independent experiments. Each independent experiment had six biological replicates. The data are expressed as the means ± SD from six biological replicates. Lowercase letters indicate significant differences among the controls and the experimental group by one-way ANOVA test in each independent experiment (*P* < 0.05).

A Y1H screening was performed to search candidate transcription factors possibly regulating *VvCCD4b* expression. The short fragment of the *VvCCD4b* promoter was used as a bait to screen the cDNA library of “Cabernet Sauvignon.” Nine candidate transcription factors were captured (Supplementary Table S5). Except for *VvPCL1*, which failed to be cloned successfully, the CDS of the other transcription factor genes was all obtained and subcloned into the pCAMBIA 1301 vector. Following that, a dual-luciferase reporter assay in a tobacco leaf transient expression system was carried out to test their influences on the activity of the *VvCCD4b* promoter (Figure 5 and Supplementary Figure S3). At least two independent experiments all demonstrated that VvbZIP53, VvMYB4, VvWRKY40-like, VvGATA24, and VvbHLH47 could not activate the *VvCCD4b* promoter, whereas VvMYBCS1 and VvMYB1R1 could suppress the activity of the *VvCCD4b* promoter only once among three independent experiments. Only VvMADS4 always showed a consistent effect on the activity of the *VvCCD4b* promoter in the four independent experiments with six biological replicates per experiment. Compared with the control group (pGreen-P*_CCD__4__b_* + pCAMBIA 1301), the relative LUC/REN ratio was significantly lower when the *LUC*-containing construction was co-expressed with pCAMBIA 1301-VvMADS4, indicating that VvMADS4 decreased *VvCCD4b* promoter activity (Figure 5). The co-expression analysis between *VvCCD4b* and the above nine transcription factors was conducted using RNA-seq data across the whole development period of grape berry^7^. The *VvMYBCS1*, *VvMYB1R1*, *VvWRKY40-like*, *VvbZIP53*, and *VvMYB4* were all negatively correlated with the *VvCCD4b* expression, and Pearson’s coefficient values were −0.66, −0.78, −0.68, −0.76, and −0.54, respectively. *VvMADS4*, *VvbHLH47*, *VvGATA24*, and *VvPCL1* were positively correlated with *VvCCD4b* expression, and Pearson’s coefficient values were 0.63, 0.20, 0.41, and 0.58, respectively. *VvMADS4* co-expressed with *VvCCD4b* with the highest coefficient. Moreover, the *VvCCD4b* promoter included a CArG box *cis*-acting element, required by MADS binding (Figure 3A). Therefore, the biological functions of VvMADS4 were further researched.

**FIGURE 6:**
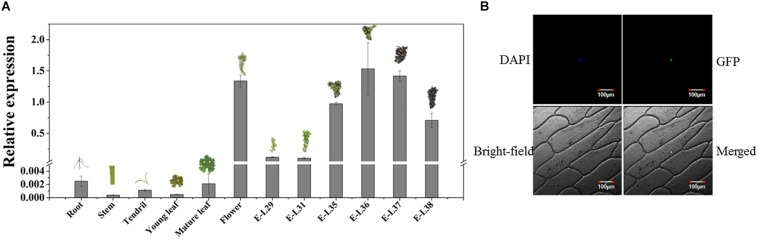
Characterization of VvMADS4. **(A)** Temporal and spatial expression patterns of *VvMADS4*. The data are expressed as means ± SD of three replications. **(B)** Subcellular localization of VvMADS4. DAPI is a nuclear-localized marker.

The temporal and spatial expression assays showed that *VvMADS4* was mainly expressed in grape flowers and berries (Figure 6A). *VvMADS4* expression increased as the grape berries developed until E-L36, after which point, it began decreasing.

Subcellular localization of VvMADS4 in onion epidermal cells revealed that VvMADS-GFP was only present in the nucleus, confirming its role as a transcription factor (Figure 6B).

**FIGURE 7:**
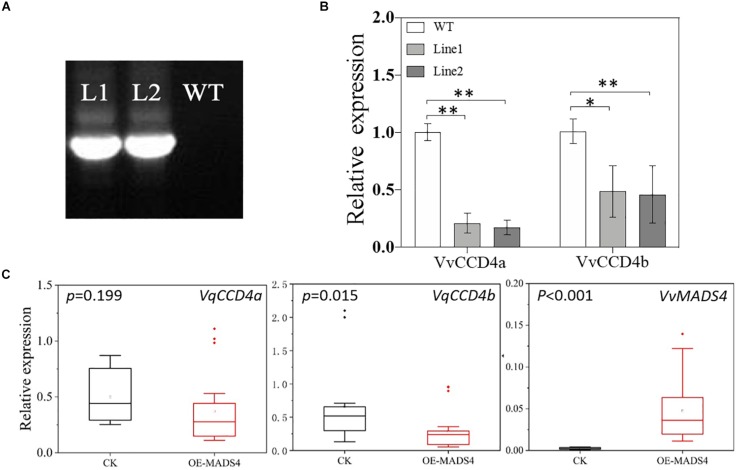
Transcriptional regulation of VvMADS4 on the *VvCCD4b* gene. **(A)** Hygromycin gene expression detected by PCR and analyzed by gel electrophoresis. The original figure is shown in Supplementary Figure S4. **(B)** Relative expression levels of *VvCCD4s* in transgenic grape calli carrying the *VvMADS4* sequence. The name of the pertinent gene is indicated on each panel. Data are expressed as means ± SD of three replicates. Asterisks indicate significant differences relative to the control by one-way ANOVA test (^∗^*P* < 0.05; ^∗∗^*P* < 0.01). **(C)** Relative expression levels of *VqCCD4s* in *VvMADS4* transiently overexpressed *V. quinquangularis* leaves. At least seven biological replicates were carried out, and a one-way ANOVA test was performed (^∗^*P* < 0.05; ^∗∗^*P* < 0.01).

To elucidate the regulatory effect of VvMADS4 on *VvCCD4b* transcription in the homologous system, we overexpressed *VvMADS4* stably in grape calli and transiently in *V. quinquangularis* leaves. It was observed that transgenic calli expressed the *Hygromycin* gene, whereas wild-type calli did not, demonstrating that the CaMV 35S promoter-driven Vv*MADS4* gene (35:*VvMADS4*) was successfully transformed into the calli (Figure 7A). In comparison to that in the wild-type calli, overexpression of *VvMADS4* resulted in the reduction of *VvCCD4b* and *VvCCD4a* expression amounts (Figure 7B). Additionally, *VvMADS4* was transiently overexpressed in *V. quinquangularis* leaves, and an empty vector was also transformed as the control. We observed significant downregulation of *VvCCD4b* in *VvMADS4*-overexpressed leaves (Figure 7C). Both of the results suggest that VvMADS4 overexpression negatively regulates *VvCCD4b* transcription.

## Discussion

### Importance of VvCCD4b in Grape Berries

CCDs are instrumental in norisoprenoid production because they cleave double bonds at specific sites in carotenoid molecules; *in vitro* and *in vivo* recombinant enzymatic experiments have yielded strong evidence for this (Rubio et al., 2008; Huang et al., 2009; Ma et al., 2013; Rodrigo et al., 2013; Rubio-Moraga et al., 2014; Bruno et al., 2015), as have *in planta* experiments (Campbell et al., 2010; Lashbrooke et al., 2013; Zhang et al., 2015). Overexpression experiments in carotenoid-accumulating *E. coli* or *S. cerevisiae* verified that VvCCD1 cleaves zeaxanthin, lycopene, ε-carotene, and β-carotene to yield 3-hydroxy-β-ionone, MHO, geranylacetone, β-ionone, and β-cyclocitral, respectively (Mathieu et al., 2005; Lashbrooke et al., 2013; Meng et al., 2019). Another study noted that overexpressing or silencing *VvCCD1* in transgenic grapevine did not influence leaf norisoprenoid levels (Lashbrooke et al., 2013). However, overexpressing *VvCCD4a* and *VvCCD4b* in carotenoid-accumulating *E. coli* revealed that VvCCD4a and VvCCD4b could cleave neurosporene, lycopene, and ε-carotene to generate geranylacetone, MHO, and α-ionone, respectively (Lashbrooke et al., 2013). Overexpressing *VvCCD4b* in β-carotene-accumulating *S. cerevisiae* resulted in the formation of β-ionone and β-cyclocitral (Meng et al., 2019). However, there is no *in planta* evidence regarding the function of VvCCD4a and VvCCD4b.

In this study, we demonstrated that *VvCCD4b* expression is significantly correlated with the accumulation of norisoprenoids, particularly MHO, β-ionone, and geranylacetone. These three compounds are all direct products of VvCCD4b interacting with carotenoids (Lashbrooke et al., 2013; Meng et al., 2019). Together, our findings and previous data strongly indicate that VvCCD4b is a critical enzyme affecting norisoprenoid production in grape berries.

### Development-Dependent V*vCCD4b* Expression

Although CCDs in different species are homologous, their expression patterns are species specific. In *Malus domestica*, *MdCCD4b* is mainly expressed in flowers but not in fruits or buds (Chen H. et al., 2018). *CCD4b* of *Solanum lycopersicum* is expressed in all tissues but is the most prominent in mature leaves and the least prominent in fruit (Wei et al., 2015). In *C. sinensis*, *CsCCD4b1* is expressed only in petals and fruit peel. In the latter, *CsCCD4b1* expression is the lowest at the green stage, peaks at the late breaker stage, and decreases until ripening (Rodrigo et al., 2013). In potato, *CCD4* is primarily expressed in leaves and flowers, with much lower expression in stems, tubers, and roots (Campbell et al., 2010). *CCD4* of *Rosa* × *damascena* is predominantly expressed in flowers; it exhibits very low expression in leaves, stems, and roots (Huang et al., 2009).

Here, our study reveals that *VvCCD4b* is abundantly expressed in mature leaves, flowers, and ripening berries, similar to what was observed in “Pinotage” (Lashbrooke et al., 2013). Notably, *VvCCD4b* expression was induced at the beginning of the coloration stage (E-L35) compared to the earlier stages. The timing of the expression, combined with our results from the ABA spraying of grapes, indicates that ABA upregulates *VvCCD4b* expression. The present findings corroborate the previous report in apples where the researchers observe that ABA induces the expression of *MdCCD4c*, *MdCCD7b*, and *MdCCD8a* (Chen H. et al., 2018). Likewise, ABA also increases *CCD4* expression in soybean (Wang et al., 2011). Combining these above observations, we suggest that the *VvCCD4b* expression promoted by ABA might be related to the ABRE element on the *VvCCD4b* promoter.

CCDs have distinct expression patterns and divergent functions. The *cis*-acting elements are not well conserved among *CCD* promoters in numerous plants, such as *A. thaliana*, *Brassica rapa*, *Crocus sativus*, *Medicago truncatula*, *Oryza sativa*, *Populus trichocarpa*, *Sorghum bicolor*, *S. lycopersicum*, and *V. vinifera* (Ahrazem et al., 2010). The present study preliminarily indicates that the activity of the *VvCCD4b* promoter is dropped in response to the 37°C treatment and that it also responds to the illumination change. This finding diverges from those of some previous reports. In soybean, cold and heat treatments both increase *CCD4* expression, though cold treatment for 6 and 12 h did decrease the expression (Wang et al., 2011). Cold and heat treatments also upregulate *CsCCD4c* expression in *C. sativus* (Rubio-Moraga et al., 2014). Exposure to red, blue, and white light strongly decreases *SbCCD4* in *Scutellaria baicalensis* (Tuan et al., 2017), whereas darkness treatment decreases *CCD4b1* expression in clementines, but not in Navelina oranges (Lado et al., 2019).

Considering the finding of the *VvCCD4b* promoter responding to different light and temperature conditions, we tried to dissect why the expression patterns of *VvCCD4b* diverged during the ripening stage in different regions and different vintages. In this study, the GT and CL regions are characterized by a temperate continental arid climate and a temperate continental monsoon climate. Under both climates, diurnal temperature difference and sunshine are responsively altered by rainfall. In our grape-producing regions, extensive field investigations also support the fact that grape berry quality is strongly affected by seasonal rainfall. The researchers also reported that the accumulation patterns of β-damascenone and TDN were correlated with precipitation and humidity and that the expression pattern of *VvCCD4b* was also influenced by water deficit (Xu et al., 2015; Savoi et al., 2016; Chen et al., 2017). Only the responses of *VvCCD4b* promoter activity to light and temperature treatments are insufficient to interpret the relevance of *VvCCD4b* expression in the production of norisoprenoids in grapes. The rainfall and water status are also important factors. More experiments need to be conducted.

### Functional Characterization of VvMADS4

In this study, we found that VvMADS4 is a potential transcription factor negatively regulating *VvCCD4b* expression. MADS transcription factors regulate fruit ripening (Ito et al., 2008), vegetative organ development (Guo et al., 2017; Li et al., 2019), flowering time (Jeon et al., 2000; Alter et al., 2016), floral meristem and organ identity (Thompson et al., 2009), stress tolerance (Guo et al., 2016), and metabolism (Lu et al., 2018; Zha et al., 2019). MADS proteins are divided into Type I and Type II based on conserved motifs and exon count. Type II (also known as MIKC) includes MADS (M-), intervening (I-), keratin-like (K-), and C-terminal (C-) domains (Theißen et al., 1996; Díaz-Riquelme et al., 2009). MIKC-type genes are classified as MIKC^*C*^- and MIKC^∗^- based on the I domain (Henschel et al., 2002). MIKC^*C*^ members can be further subdivided into A, B, C, D, and E classes according to their function in flower organogenesis (Theißen and Saedler, 2001). VvMADS4 belongs to the E-class and is homologous with *AtSEP3* from *Arabidopsis* (Boss et al., 2002; Wang L. et al., 2015; Grimplet et al., 2016). AtSEP3 interacts with other MADS to influence flower development and organ identity (Immink et al., 2009; Melzer et al., 2009; Wu et al., 2012). Specifically, AtSEP3 binds to LEAFY to activate B- and C-class genes (Krizek and Fletcher, 2005; Liu et al., 2009). This pair also directly activates AP3, AG, SEP1-4, and AP1 (Kaufmann et al., 2009) and is part of the positive feedback loop that maintains ABCE gene expression (Liu and Mara, 2010). AGAMOUS-like24 (AGL24), SUPPRESSOR OF OVEREXPRESSION OF CO1 (SOC1), and SHORT VEGETATIVE PHASE (SVP) all repress *AtSEP3* expression (Gregis et al., 2008; Kaufmann et al., 2009). SEPs are involved in regulating fruit ripening in fleshy fruits. For example, the best-known ripening-related gene in tomato is *Rin*, a *SEP4*-like gene and MADS family member (Vrebalov et al., 2002). Likewise, FaMADS9, a member of the SEP1/2 subfamily, modulates strawberry receptacle, achene, and petal development (Seymour et al., 2011). MaMADS2, a SEP3 homolog in banana, increases ethylene production; however, the un-ripening phenotype is still not complemented (Elitzur et al., 2010). Similarly, in the *VvMADS4*-transformed tomato *rin* mutant, the un-ripening phenotype is not complemented (Mellway and Lund, 2013). Collectively, all of these results indicate that MADS transcription factors participate in fruit ripening development by interacting with each other.

MADS proteins contact DNA by inserting amino acid residues of the α-helix in the N-terminus into the major groove or into the minor groove of the DNA (Pellegrini et al., 1995; Huang et al., 2000; Santelli and Richmond, 2000). There is a conserved arginine residue at the third amino acid position (R3) in the N-terminal arm of the MADS domain, which is directed into the minor groove of the DNA (Pellegrini et al., 1995; Huang et al., 2000; Santelli and Richmond, 2000). Using a lysine or an alanine residue to replace the R3 will reduce the DNA-binding affinity (Käppel et al., 2018). The VvMADS4 binding domain that interacts with the *VvCCD4b* promoter was not identified in this study. Although VvMADS4 was firstly screened out by a Y1H screening assay, when we verified their interaction again by the Y1H system using the *VvCCD4b* promoter sequence as the bait, the unexpected result was attained that VvMADS4 could not bind with the promoter (Supplementary Figure S5). Further study found that the VvMADS4 could bind to the three-tandem CArG box sequence, which was designed with reference to the sequence of CArG box on the *VvCCD4b* promoter. The number of binding sites may influence the binding efficiency. SEP3 specifically binds to CArG box [CC(A/T)_6_GG], whereas CArG box [CAAATTTAAG] was not consistently found in the *VvCCD4b* promoter, which may be also a reason for the low binding efficiency. MADS proteins usually bind to the CArG motif of the target gene DNA as dimers or multiple complex (Schwarzsommer et al., 1992; Shore and Sharrocks, 1996). However, not all the CArG elements combine with MADS proteins. Previous researchers found that the promoter of *VERDANDI* (*VDD*) contained three different CArG elements and that SEEDSTICK (STK) and SEP3 preferred to bind to CArG box 1 and CArG box 3 in the *VDD* promoter; as a result, STK-SEP3 protein–protein cooperative interactions form a loop between CArG boxes 1 and 3. CArG box 2 was ignored in the normal condition. But, when there was a mutant of CArG box 1 or CArG box 3, the multiple complex of STK and SEP3 could bind to CArG box 2 and another normal CArG box 1 or 3. When both CArG box 1 and CArG box 3 were mutant, the STK–SEP3 interactions could not bind to CArG box 2 (Matias-Hernandez et al., 2010; Mendes et al., 2013). So it is thought that the preference of the CArG box sequence and the number of CArG box are all important for MADS binding with DNA. SEP always coordinates with AG, SEP, and AP1. Proteins that interact with VvMADS4 (VvSEP3) in grape berries at veraison have been screened previously (Mellway and Lund, 2013). VvMADS4 can form a binary complex with VvAP3.2, VvAG1, VvAG2, VvSEP3, and VvSEP4. It can also form a ternary complex with VvAG1 and VvAG1; VvAG1 and VvAG2; or VvAG1 and VvAG3. In transgenic tomatoes, VvSEP4, VvAG1, and VvAG2 are all involved in regulating carotenoid metabolism. In our study, a dual-luciferase activity assay in *Arabidopsis* protoplast also revealed that VvMADS4 upregulated *VvCCD4b* promoter activity, which is opposite to the results in the tobacco and grape systems (Supplementary Figure S6). Therefore, we propose that VvMADS4 regulates *VvCCD4b* expression by coordinating with other proteins, in addition to binding directly to the *VvCCD4b* promoter. Future studies should directly test this hypothesis.

In summary, we verified that *VvCCD4b* expression was positively correlated with norisoprenoid accumulation in developing grape berries; we also investigated the responses of the *VvCCD4b* promoter to high temperatures and different illuminations. Furthermore, this study indicated that *VvCCD4b* expression was induced by ABA and that VvMADS4, a nucleus-localized transcription factor, possessed a potential function in downregulating *VvCCD4b* expression. Both *VvMADS4* and *VvCCD4b* were mainly expressed in flowers and ripening berries undergoing isoprene metabolism. Our findings may be valuable for efforts to improve grape fragrance by manipulating the norisoprenoid content. Moreover, this study provides insight into *VvCCD4b* expression regulation. In the future, we aim to screen for potential co-regulators of *VvCCD4b* that interact with VvMADS4. We will also try to establish *VvMADS4* transgenic grapevines, which should allow us to clarify the transcription factor’s effects on norisoprenoid accumulation.

## Data Availability Statement

This study used publicly available datasets, available at https://www.ncbi.nlm.nih.gov/geo/query/acc.cgi?acc=GSE129916.

## Author Contributions

NM, YG, and YW performed the research and analyzed data. KY, JC, and X-YL perfected the research scheme. C-QD and Q-HP guided the research. NM wrote the paper. All the authors critically revised the manuscript.

## Conflict of Interest

The authors declare that the research was conducted in the absence of any commercial or financial relationships that could be construed as a potential conflict of interest.

## Footnotes

1 http://genomes.cribi.unipd.it/grape/

2 https://www.dna.affrc.go.jp/PLACE/?action=newplace

3 http://bioinformatics.psb.ugent.be/webtools/plantcare/html/

4 http://linux1.softberry.com/berry.phtml?topic=tssp&group=programs&subgroup=promoter

5 https://deepgreen.dpb.carnegiescience.edu/cell%20imaging%20site%20/html/vectors.html

6 https://www.ncbi.nlm.nih.gov/geo/query/acc.cgi?acc=GSE129916

7 https://www.ncbi.nlm.nih.gov/geo/query/acc.cgi?acc=GSE129916
