# Supplementary material for: Characterization of Transcriptional Expression and Regulation of Carotenoid Cleavage Dioxygenase 4b in Grapes
Source: Front Plant Sci. 2020 May 8;11:483. doi: 10.3389/fpls.2020.00483 (PMC7227400; doi:10.3389/fpls.2020.00483)
Supplement: Supplementary file 1 [file Data_Sheet_1.docx]

Supplementary Material

Table S1. Primers used in this study

| **Primer name** | **Size of amplicons** | **Forward/Reverse primer sequence (5’-3’)** |
| --- | --- | --- |
| **qRT-PCR (10 µM)** |  |  |
| VvCCD1-F | 73 bp | AGCTACACTGGCAGGAAACAAC |
| VvCCD1-R |  | TAATGCCCGTCACCTTGGCTATG |
| VvCCD4a-F | 73 bp | TGAAGCTGGACGTGTCCCAAAC |
| VvCCD4a-R |  | TAGCACCCTGGTCCATACATCCTG |
| VvCCD4b-F | 100 bp | GTTGGAATCATCCCTCGTT |
| VvCCD4b-R |  | CCTCGTCCCAGGCGTTA |
| VvMADS4-F | 257 bp | TGAAGCAGATCAGGTCAACGC |
| VvMADS4-R |  | ATCGGGCTGGTATCCGATTTG |
| VvUbiquitin-F | 105 bp | CAGCCTTCTGGTAAACATAGGTGAG |
| VvUbiquitin-R |  | AGGAGTGTCCGAATGCTGAGTG |
| **RT-PCR (10 µM)** |  |  |
| AtActin8-F* | 320 bp | GCTGAGAGATTCAGGTGCCC |
| AtActin8-R |  | GAGATCCACATCTGCTGG |
| GUS-F | 255 bp | TAATGTTCTGCGACGCTCAC |
| GUS-R |  | CCAGCCATGCACACTGATAC |
| **Gene cloning (10 µM)** |  |  |
| P_CCD4b_-F | 1055 bp | TCACGACTTTATTGTTTCCC |
| P_CCD4b_-R |  | AAAGGTGGAGTGGAGTTTAT |
| T-VvMADS4-F | 729 bp | ATGGGGAGAGGTAGGGTTGA |
| T-VvMADS4-R |  | TCATGGCAACCATCCCGGCA |
| T-VvMYBCS1-F | 963 bp | ATGAGAAATCCGGCATCTGC |
| T-Vv MYBCS1-R |  | TCAGGGAGACATGGAGTGTT |
| T-VvMYB1R1-F | 903 bp | ATGTCTCGTTCCTGCTCTCA |
| T-Vv MYB1R1-R |  | TTACGCGACACTAATGATGT |
| T-VvPCL1-F | 936 bp | ATGGGCGAAGAAGTGAGGA |
| T-Vv PCL1-R |  | TTATTGATCATTAGGACCGA |
| T-VvbHLH47-F | 735 bp | ATGGTTTCGGAGGTTCCTTC |
| T-Vv bHLH47-R |  | TTAGTCTCCCCTGCTGCTGG |
| T-VvWRKY4-F | 936 bp | ATGCCTATGGATAGTTCTAAT |
| T-VvWRKY4-R |  | TCACCATTTTTCAGTTTGAT |
| T-VvbZIP-F | 438 bp | ATGGGTGGCCCAAGGCAAAC |
| T-VvbZIP-R |  | TCAGAACTGCAACATGTCTGA |
| T-VvGATA24-F | 900 bp | ATGGATGGAATCCATGGAAA |
| T-VvGATA24-R |  | TCACGCTTGATCACTAACAT |
| T-VvMYB4-F | 618 bp | ATGGTGAGGACTCCTTGTTG |
| T-VvMYB4-R |  | CTAGGTAGTTTCTCTCTCGC |
| **Promoter activity analysis (10 µM)** |  |  |
| pCambia1300-P_CCD4b_-LUC-F | 1092 bp | GGAAATTCGAGCTCGGTACTCACGACTTTATTGTTTCCC |
| pCambia1300-P_CCD4b_-LUC-R |  | GCAGATCTCGAGCCCGGGAAAGGTGGAGTGGAGTTTAT |
| **Transgenic *A. thaliana* construct (10 µM)** |  |  |
| pCambia1381-P_CCD4b_-GUS-F | 1086 bp | CGCCGAATTCCCGGGTCACGACTTTATTGTTTCCC |
| pCambia1381-P_CCD4b_-GUS-F |  | TTGGCTGCAGGTCGACAAAGGTGGAGTGGAGTTTAT |
| **Y1H screening assay (10 µM)** |  |  |
| pAbAi-P_VvCCD4b_-F | 688 bp | TCACGACTTTATTGTTTCCC |
| pAbAi-P_VvCCD4b_-R |  | TATATGGGCCGTTTGTTCATCTTCC |
| pGADT7-F |  | TAATACGACTCACTATAGGGC |
| pGADT7-R |  | AGATGGTGCACGATGCACAG |
| **Subcellular localization (10 µM)** |  |  |
| pEZS-NL-VvMADS4-F | 770 bp | TTTGGAGAGGACACGCTCGAGATGGGGAGAGGTAGGGTTGA |
| pEZS-NL-VvMADS4-R |  | GGATCCCGGGCCCGCGGTACCCTTGGCAACCATCCCGGCA |
| **Dual luciferase assay (10 µM)** |  |  |
| pGreen II 0800-P_CCD4b_-F | 912 bp | AAAACTGCAGTCACGACTTTATTGTTTCCC |
| pGreen II 0800-P_CCD4b_-R |  | CGCGGATCCTTCCTTGCCTTCCACTCTGCCT |
| pCambia1301-VvMADS4-F | 746 bp | CTAGTCTAGAATGGGGAGAGGTAGGGTTGA |
| pCambia1301-VvMADS4-R |  | CGAGCTCTCATGGCAACCATCCCGGCA |
| pCambia1301-VvMYBCS1-F | 980 bp | CTAGTCTAGAATGAGAAATCCGGCATCTGC |
| pCambia1301-Vv MYBCS1-R |  | CGAGCTCTCAGGGAGACATGGAGTGTT |
| pCambia1301-VvMYB1R1-F | 920 bp | CTAGTCTAGAATGTCTCGTTCCTGCTCTCA |
| pCambia1301-Vv MYB1R1-R |  | CGAGCTCTTACGCGACACTAATGATGT |
| pCambia1301-VvbHLH47-F | 752 bp | CGCGGATCCATGGTTTCGGAGGTTCCTTCA |
| pCambia1301-Vv bHLH47-R |  | CGAGCTCTTAGTCTCCCCTGCTGCTGG |
| pCambia1301-VvWRKY40-like-F | 953 bp | CTAGTCTAGAATGCCTATGGATAGTTCTAA |
| pCambia1301-VvWRKY40-like-R |  | CGAGCTCTCACCATTTTTCAGTTTGAT |
| pCambia1301-VvbZIP-F | 455 bp | CTAGTCTAGAATGGGTGGCCCAAGGCAAAC |
| pCambia1301-VvbZIP-R |  | CGAGCTCTCAGAACTGCAACATGTCTG |
| pCambia1301-VvGATA24-F | 917 bp | CTAGTCTAGAATGGATGGAATCCATGGAAA |
| pCambia1301-VvGATA24-R |  | CGAGCTCTCACGCTTGATCACTAACAT |
| pCambia1301-VvMYB4-F | 635 bp | CTAGTCTAGAATGGTGAGGACTCCTTGTTG |
| pCambia1301-VvMYB4-R |  | CGAGCTCCTAGGTAGTTTCTCTCTCGC |
| **Overexpression construct in callus (10 µM)** |  |  |
| pCXSN-VvMADS4-F | 771 bp | GGGAAATTCGCTAGTGGATCCATGGGGAGAGGTAGGGTTGA |
| pCXSN-VvMADS4-R |  | AACGATACTCGAGGGGGATCCTCATGGCAACCATCCCGGCA |
| **HygromycinB detection (10 µM)** |  |  |
| Hyg-F | 1026 bp | ATGAAAAAGCCTGAACTCACCG |
| Hyg-R |  | CTATTTCTTTGCCCTCGGACGA |

*AtAcin8-F/R, GUS-F/R were the primers of AtACT8 and GUS referred to Tanabe N, Tamoi M , Shigeoka S . The sweet potato RbcS gene (IbRbcS1) promoter confers high-level and green tissue-specific expression of the GUS reporter gene in transgenic Arabidopsis. Gene, 2015, 567(2):244-250.

Table S2. The norisoprenoids detected in grapes from Changli (CL) and Gaotai (GT) in two years

|  | **CL region** | | | |  | **GT region** | | | |
| --- | --- | --- | --- | --- | --- | --- | --- | --- | --- |
|  | **2013** | | **2016** | |  | **2013** | | **2016** | |
| **Compounds** | Free-form | Bound-form | Free-form | Bound-form |  | Free-form | Bound-form | Free-form | Bound-form |
| 6-methyl-5-hepten-2-one | √ |  | √ |  |  | √ |  | √ |  |
| TCH | √ |  | √ |  |  | √ |  | √ |  |
| *cis*-theaspirane | √ |  | √ |  |  | √ |  | √ |  |
| *trans*-theaspirane | √ |  | √ |  |  | √ |  | √ |  |
| Dihydroedulan I | √ |  | √ |  |  | √ |  | √ |  |
| VitispiraneA | √ | √ | √ | √ |  | √ | √ | √ | √ |
| VitispiraneB | √ | √ | √ | √ |  | √ | √ | √ | √ |
| *β*-cylcocitral | √ |  | √ |  |  | √ |  | √ |  |
| Riesling acetal | √ |  | √ |  |  | √ |  | √ |  |
| TDN | √ | √ | √ | √ |  | √ | √ | √ | √ |
| *β*-damascenone*-E* | √ | √ | √ | √ |  | √ | √ | √ | √ |
| *β*-damascenone*-Z* | √ | √ | √ | √ |  | √ | √ | √ | √ |
| Geranylacetone | √ |  | √ |  |  | √ |  | √ |  |
| *β*-ionone | √ |  | √ |  |  | √ |  | √ |  |
| TPB | √ | √ | √ | √ |  | √ | √ | √ | √ |

Table S3. The concentration of norisoprenoids in grapes from Changli (CL) and Gaotai (GT) in 2013 and 2016 (unit: μg/berry).

| Sample name^a^ | 6-methyl-5-hepten-2-one | TCH | *Cis*-theaspirane | dihydroedulan I | Vitispirane A | Vitispirane B | *β*-cyclocitral | Riesling acetal | TDN | *β*-damascenone-*E* | geranylacetone | *β*-ionone | *Trans*-theaspirane | TPB | *β*-damascenone-*Z* | Total |
| --- | --- | --- | --- | --- | --- | --- | --- | --- | --- | --- | --- | --- | --- | --- | --- | --- |
| CL 2016 E-L31 | 0.76±0.21^b^ | 0.12±0.03 | 0.09±0.01 | 0.01±0.00 | 4.14±0.31 | 1.94±0.09 | 0.06±0.01 | 0.15±0.01 | 13.14±0.90 | 18.40±0.59 | 0.33±0.03 | 0.06±0.01 | 0.12±0.00 | 0.14±0.01 | 1.97±0.02 | 41.55±2.23 |
| CL 2016 E-L35 | 0.59±0.01 | 0.11±0.00 | 0.41±0.01 | 0.03±0.00 | 9.62±0.58 | 4.91±0.29 | 0.13±0.01 | 0.44±0.05 | 21.29±0.61 | 35.42±0.67 | 0.46±0.00 | 0.17±0.01 | 0.54±0.02 | 0.36±0.11 | 3.40±0.06 | 78.42±2.44 |
| CL 2016 E-L37 | 1.04±0.06 | 0.09±0.01 | 0.12±0.01 | 0.10±0.00 | 8.25±0.12 | 4.41±0.04 | 0.10±0.00 | 0.09±0.00 | 42.80±0.52 | 51.66±0.91 | 0.85±0.01 | 0.24±0.02 | 0.20±0.02 | 0.64±0.02 | 5.28±0.17 | 116.07±1.93 |
| CL 2016 E-L38 | 0.91±0.20 | 0.08±0.01 | 0.02±0.00 | 0.06±0.00 | 9.44±0.88 | 5.14±0.54 | 0.06±0.01 | 0.02±0.01 | 62.02±7.61 | 54.92±4.76 | 0.98±0.03 | 0.19±0.02 | 0.03±0.01 | 0.97±0.12 | 6.15±0.47 | 141.03±14.67 |
| GT 2016 E-L31 | 0.20±0.03 | 0.06±0.00 | 0.04±0.00 | 0.00±0.00 | 0.65±0.00 | 0.28±0.00 | 0.02±0.00 | 0.02±0.01 | 3.87±1.02 | 5.56±0.02 | 0.16±0.01 | 0.03±0.01 | 0.05±0.01 | 0.02±0.00 | 0.77±0.01 | 11.75±0.02 |
| GT 2016 E-L35 | 0.63±0.02 | 0.12±0.01 | 0.20±0.01 | 0.02±0.00 | 5.28±0.15 | 2.36±0.03 | 0.12±0.01 | 0.30±0.04 | 17.06±2.29 | 26.06±0.90 | 0.45±0.01 | 0.18±0.02 | 0.27±0.02 | 0.17±0.03 | 2.80±0.13 | 56.00±1.74 |
| GT 2016 E-L37 | 0.85±0.09 | 0.08±0.00 | 0.20±0.02 | 0.10±0.02 | 3.66±0.09 | 2.08±0.02 | 0.12±0.01 | 0.07±0.02 | 22.74±7.22 | 42.57±0.57 | 0.87±0.05 | 0.30±0.06 | 0.22±0.02 | 0.31±0.01 | 4.93±0.07 | 79.10±1.00 |
| GT 2016 E-L38 | 0.67±0.00 | 0.09±0.00 | 0.15±0.00 | 0.07±0.00 | 7.17±1.54 | 3.84±0.78 | 0.12±0.00 | 0.13±0.01 | 36.19±3.37 | 51.31±4.48 | 0.75±0.00 | 0.26±0.01 | 0.20±0.01 | 0.40±0.11 | 5.43±0.35 | 106.77±13.96 |
| CL 2013 E-L34 | 0.40±0.10 | 0.04±0.01 | 0.15±0.04 | 0.00±0.00 | 2.52±0.15 | 1.02±0.07 | 0.05±0.01 | 0.10±0.03 | 8.68±0.22 | 11.37±0.15 | 0.29±0.01 | 0.06±0.02 | 0.20±0.05 | 0.07±0.01 | 1.52±0.01 | 26.48±0.40 |
| CL 2013 E-L35 | 0.62±0.10 | 0.07±0.02 | 0.31±0.07 | 0.03±0.01 | 7.65±1.29 | 3.56±0.55 | 0.10±0.02 | 0.16±0.04 | 20.14±2.26 | 24.08±3.01 | 0.43±0.03 | 0.19±0.04 | 0.36±0.08 | 0.14±0.03 | 2.59±0.19 | 60.42±7.74 |
| CL 2013 E-L36 | 0.47±0.13 | 0.04±0.02 | 0.11±0.03 | 0.00±0.00 | 4.95±0.26 | 1.94±0.07 | 0.05±0.02 | 0.12±0.03 | 12.15±1.87 | 14.11±1.12 | 0.29±0.02 | 0.07±0.01 | 0.15±0.04 | 0.12±0.01 | 1.71±0.06 | 36.29±3.09 |
| CL 2013 E-L37 | 0.71±0.14 | 0.04±0.01 | 0.41±0.05 | 0.07±0.01 | 11.89±0.76 | 6.40±0.59 | 0.10±0.03 | 0.18±0.07 | 35.52±1.98 | 43.22±9.43 | 0.75±0.14 | 0.28±0.08 | 0.49±0.07 | 0.61±0.09 | 4.49±0.59 | 105.15±14.06 |
| CL 2013 E-L38 | 0.80±0.00 | 0.03±.00 | 0.23±0.04 | 0.08±0.01 | 5.87±0.15 | 2.93±0.06 | 0.08±0.01 | 0.02±0.02 | 26.37±0.967 | 31.91±1.11 | 0.70±0.00 | 0.27±0.02 | 0.25±0.04 | 0.25±0.01 | 3.88±0.10 | 73.68±2.42 |
| GT 2013 E-L34 | 0.83±0.00 | 0.23±0.01 | 0.01±0.00 | 0.00±0.00 | 0.91±0.06 | 0.40±0.02 | 0.11±0.01 | 0.05±0.00 | 4.35±0.52 | 3.95±0.25 | 0.19±0.00 | 0.08±0,01 | 0.01±0.00 | 0.03±0.01 | 0.71±0.03 | 11.86±0.85 |
| GT 2013 E-L35 | 0.71±0.04 | 0.14±0.01 | 0.02±0.00 | 0.00±0.00 | 1.55±0.03 | 0.77±0.01 | 0.08±0.01 | 0.08±0.00 | 5.32±0.16 | 3.88±0.03 | 0.18±0.01 | 0.08±0.02 | 0.03±0.00 | 0.03±0.00 | 0.64±0.00 | 13.50±0.22 |
| GT 2013 E-L36 | 1.31±0.10 | 0.37±0.01 | 0.25±0.01 | 0.04±0.01 | 13.07±0.77 | 7.14±0.49 | 0.33±0.02 | 0.56±0.05 | 37.99±1.06 | 21.09±1.66 | 0.43±0.01 | 0.31±0.03 | 0.34±0.02 | 0.36±0.00 | 2.45±0.13 | 86.04±1.73 |
| GT 2013 E-L37 | 1.67±0.19 | 0.44±0.04 | 0.14±0.01 | 0.02±0.01 | 5.20±0.07 | 3.08±0.04 | 0.35±0.01 | 0.11±0.00 | 21.67±0.21 | 33.41±0.26 | 0.74±0.03 | 0.61±0.01 | 0.14±0.00 | 0.32±0.00 | 3.93±0.02 | 71.82±0.33 |
| GT 2013 E-L38 | 1.56±0.06 | 0.33±0.02 | 0.09±0.01 | 0.04±0.00 | 3.42±0.47 | 2.09±0.27 | 0.35±0.02 | 0.03±0.01 | 13.31±1.07 | 26.99±2.41 | 0.57±0.01 | 0.51±0.06 | 0.09±0.01 | 0.19±0.03 | 3.31±0.16 | 52.88±4.62 |

^a^ Sample name: region-vintage-E-L stage.

^b^ The concentration shown in this table is the sum of the free-form and bound-form concentration of each compound. The data are expressed as means ± SD from three biological replicates with two technical replicates, respectively. SD is standard deviation.


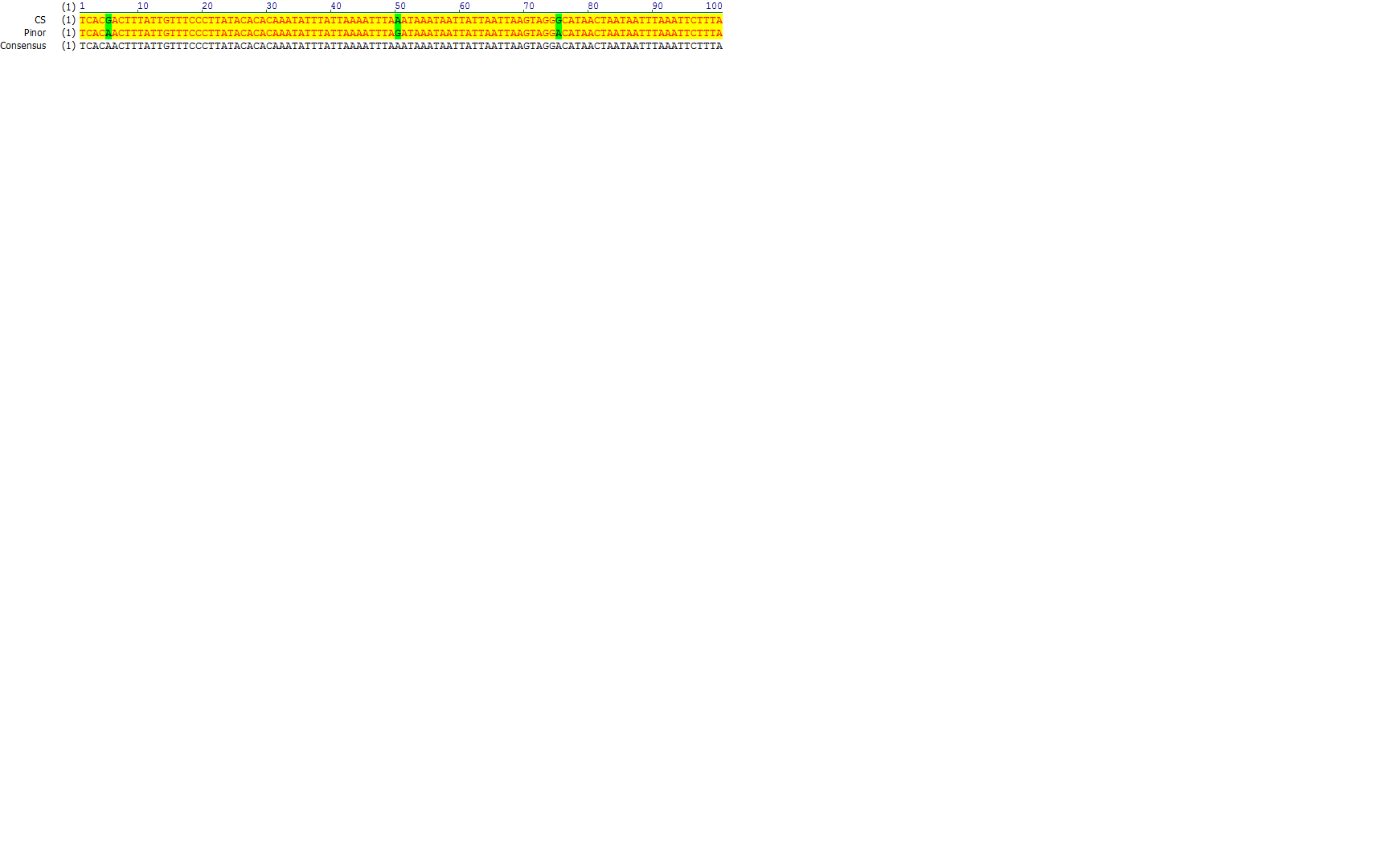

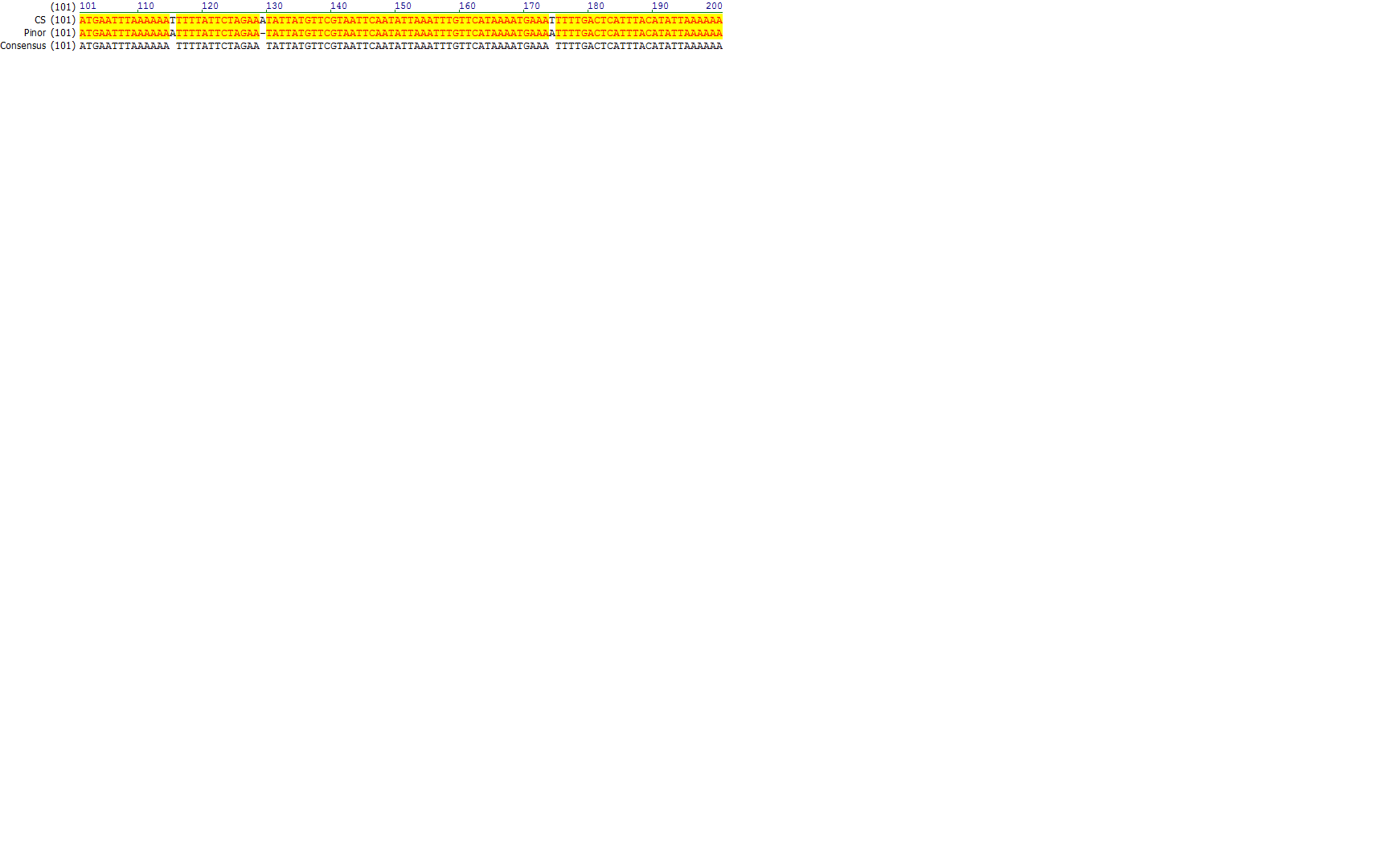

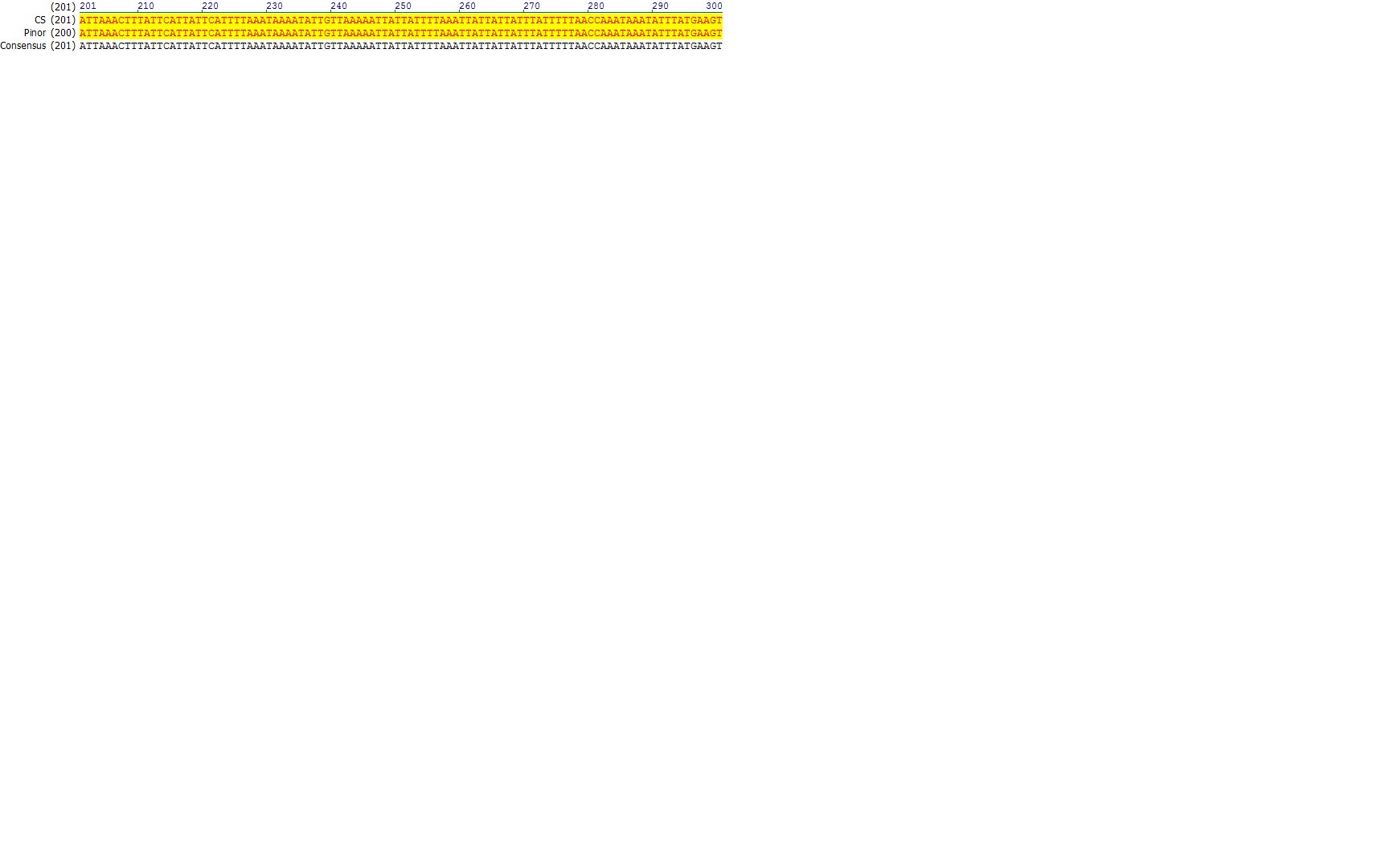

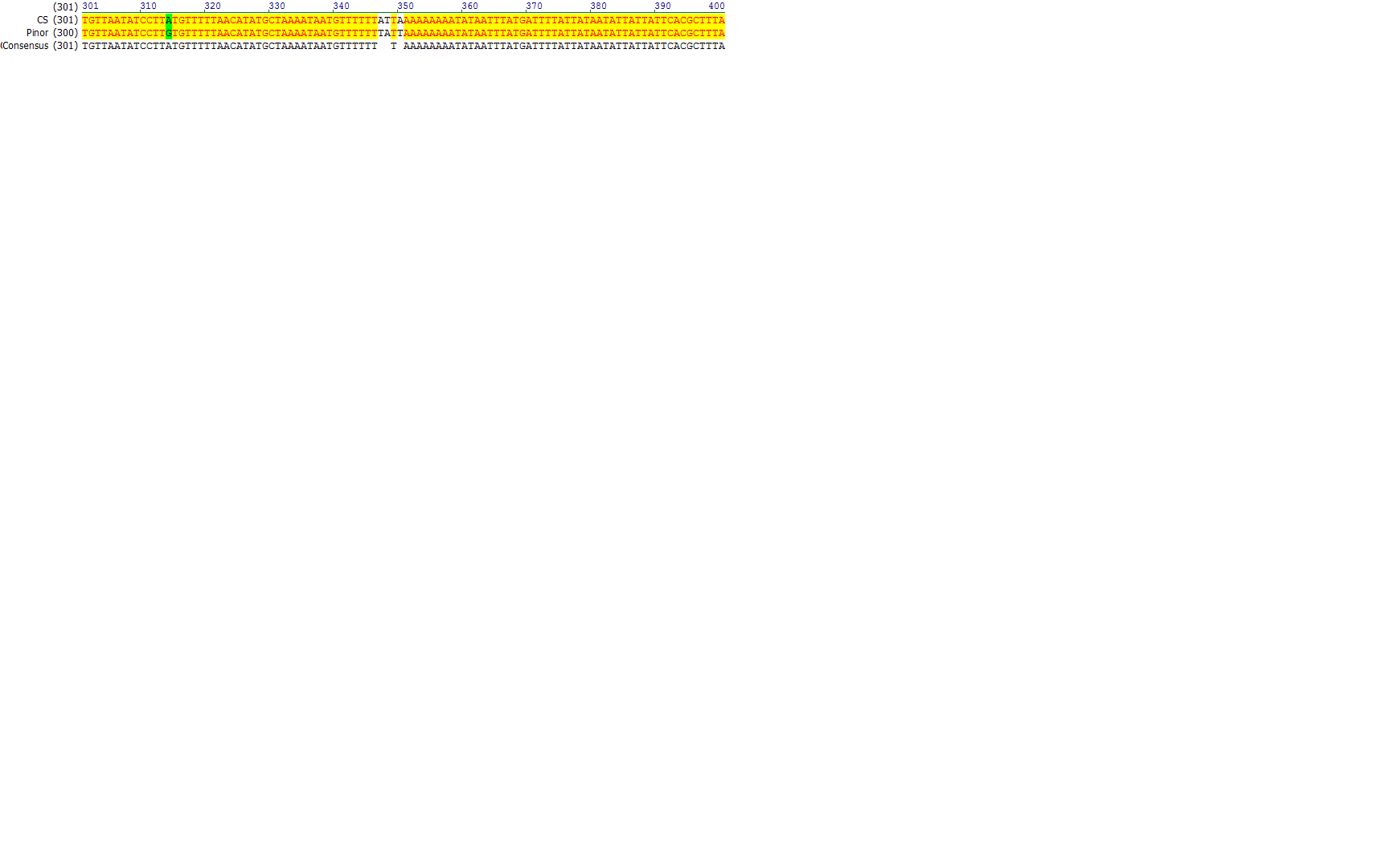

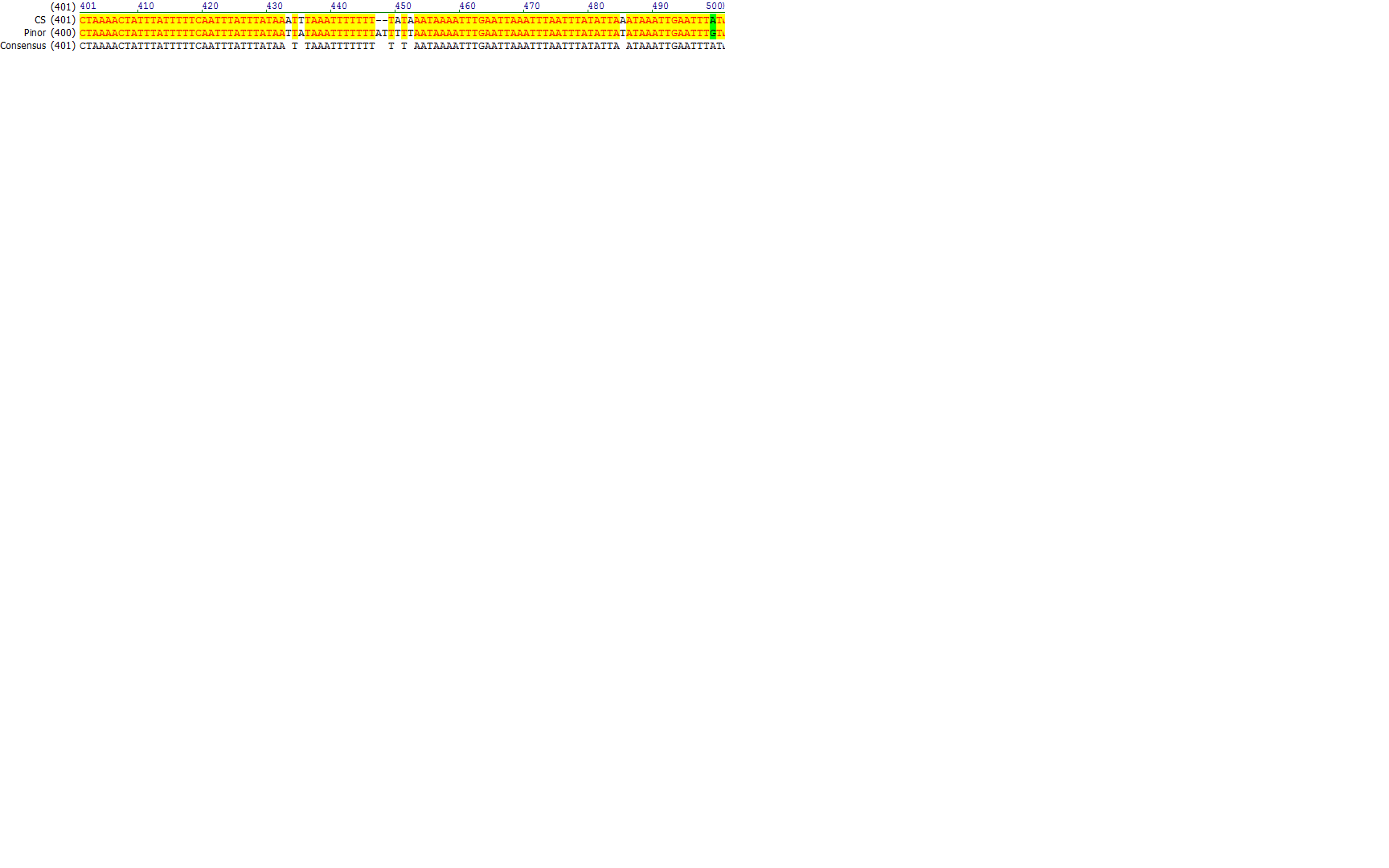

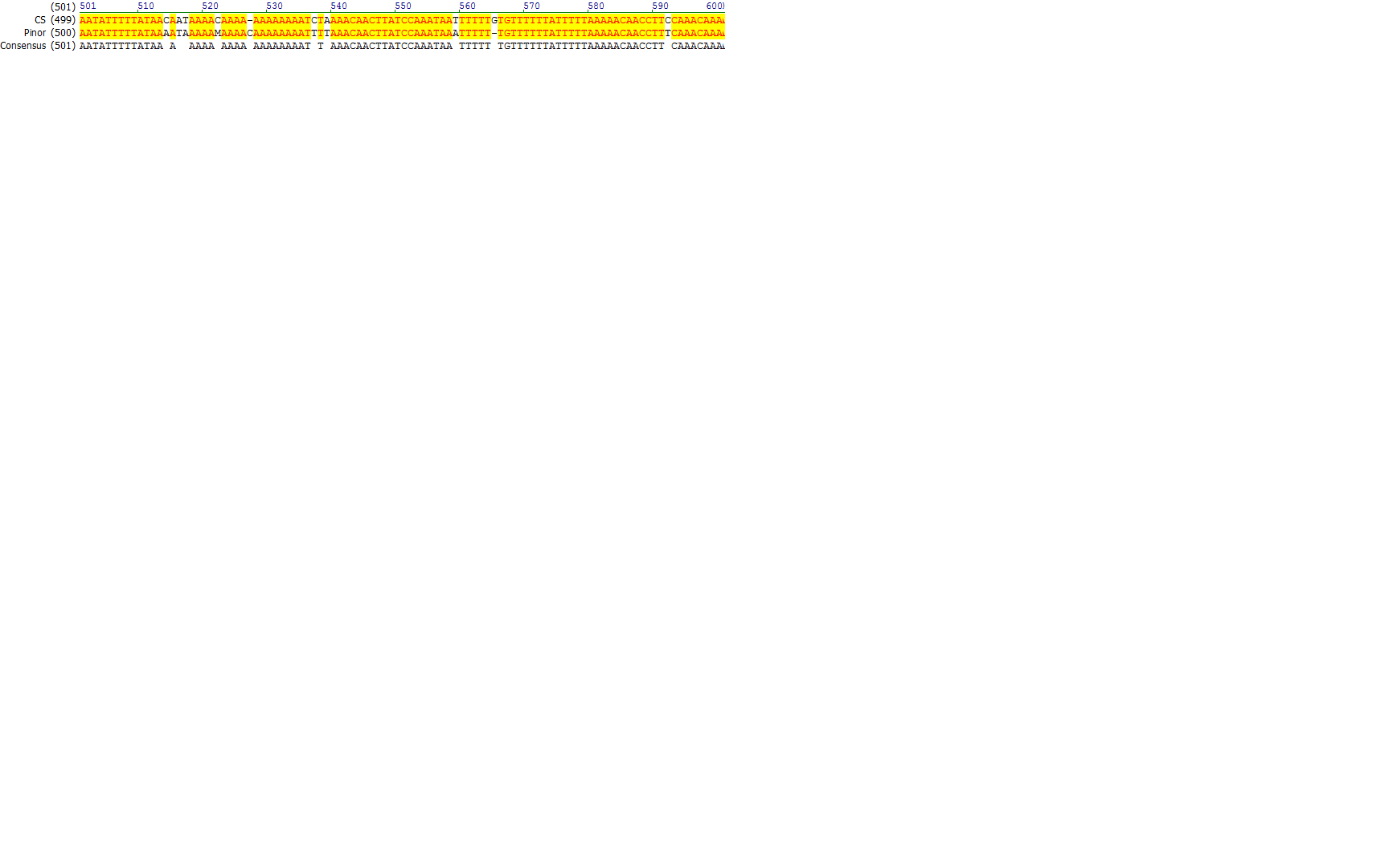

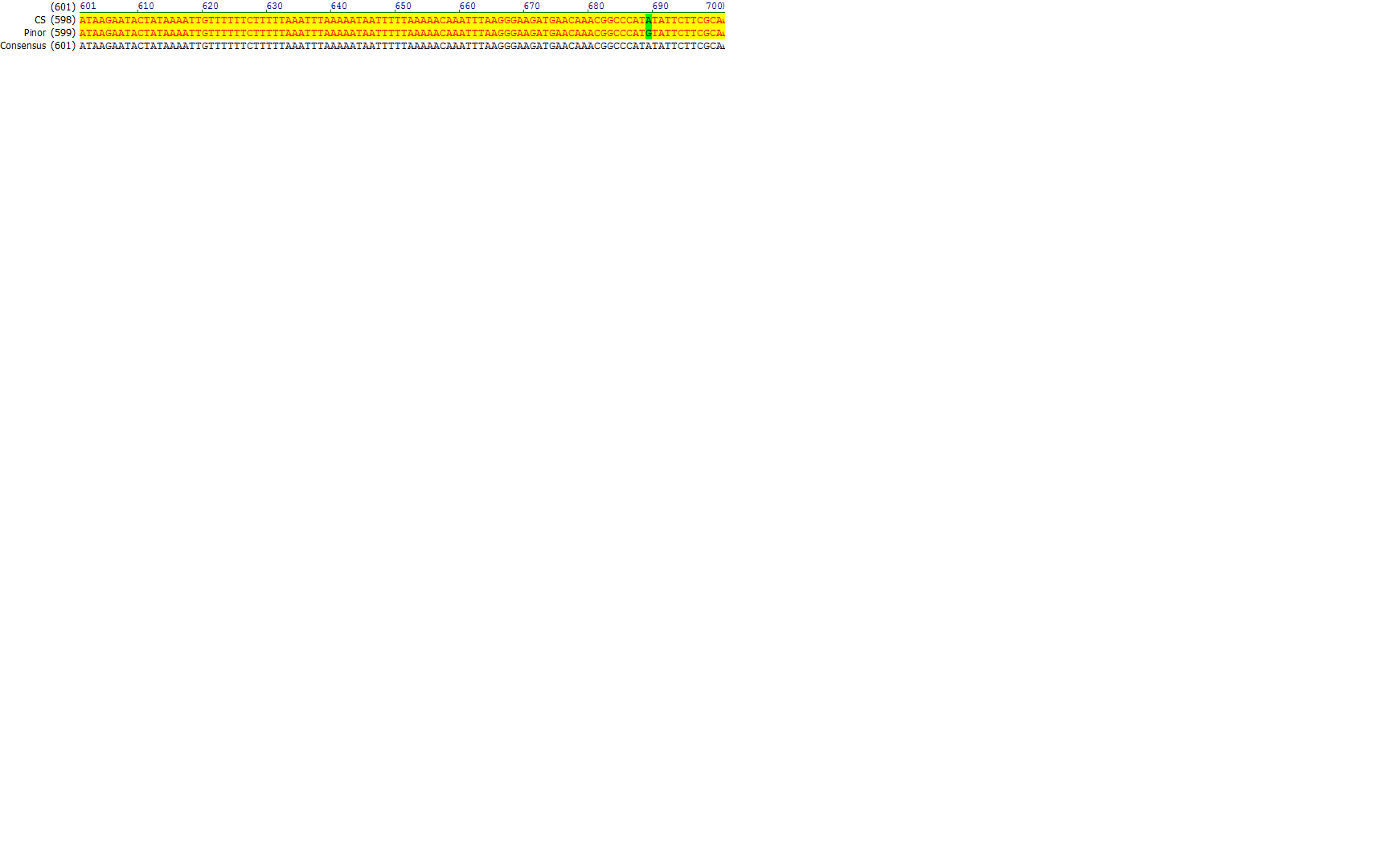

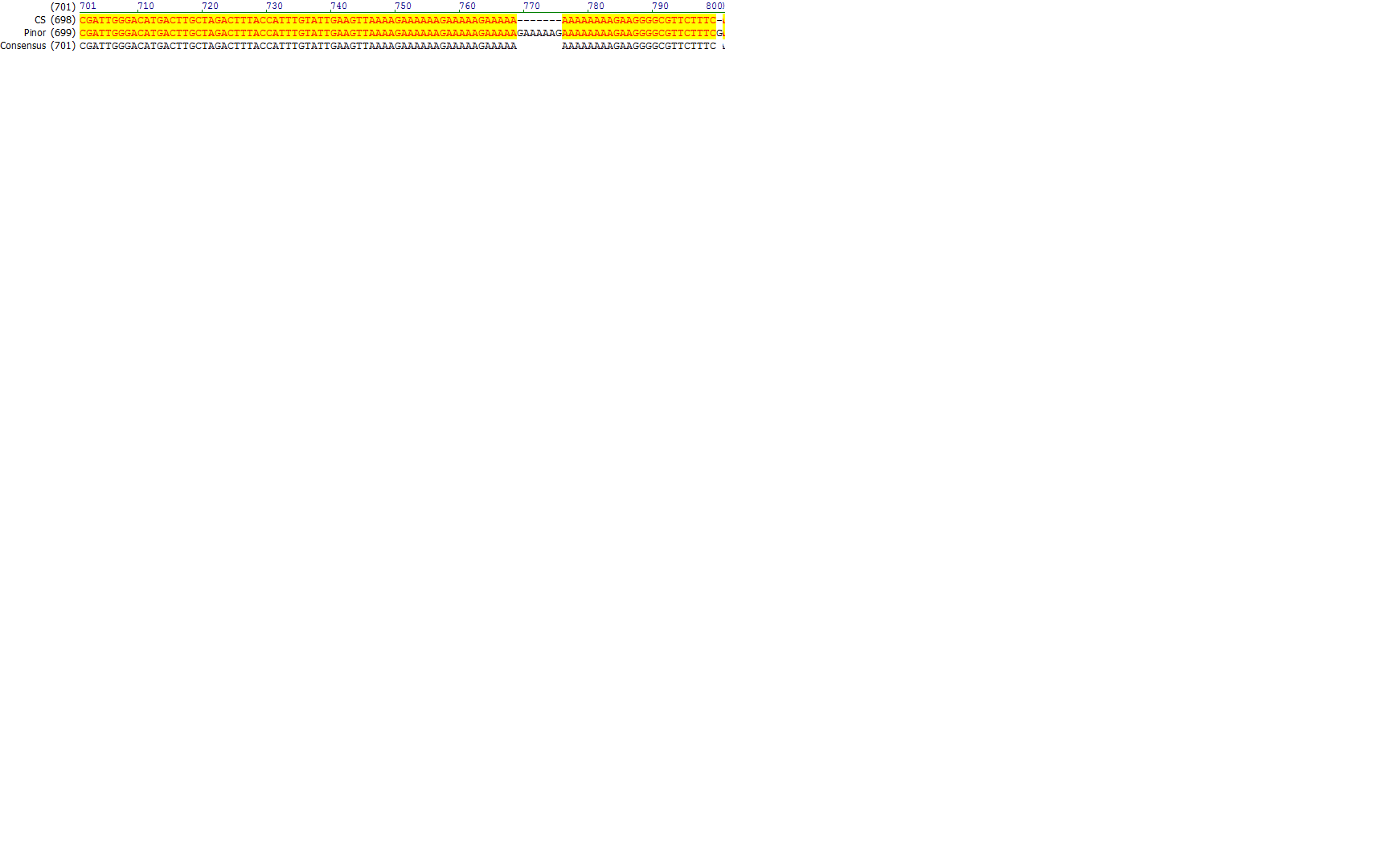

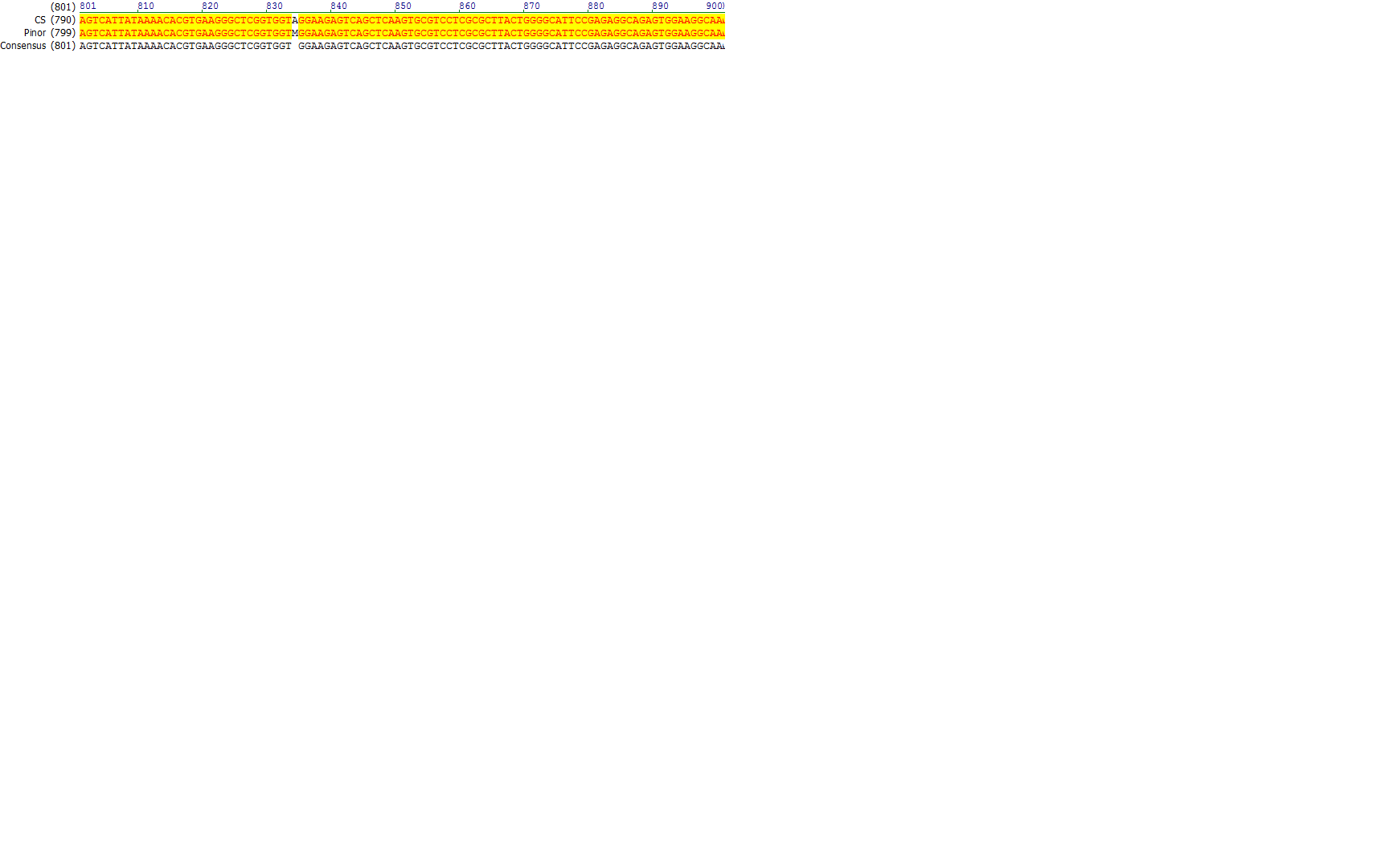

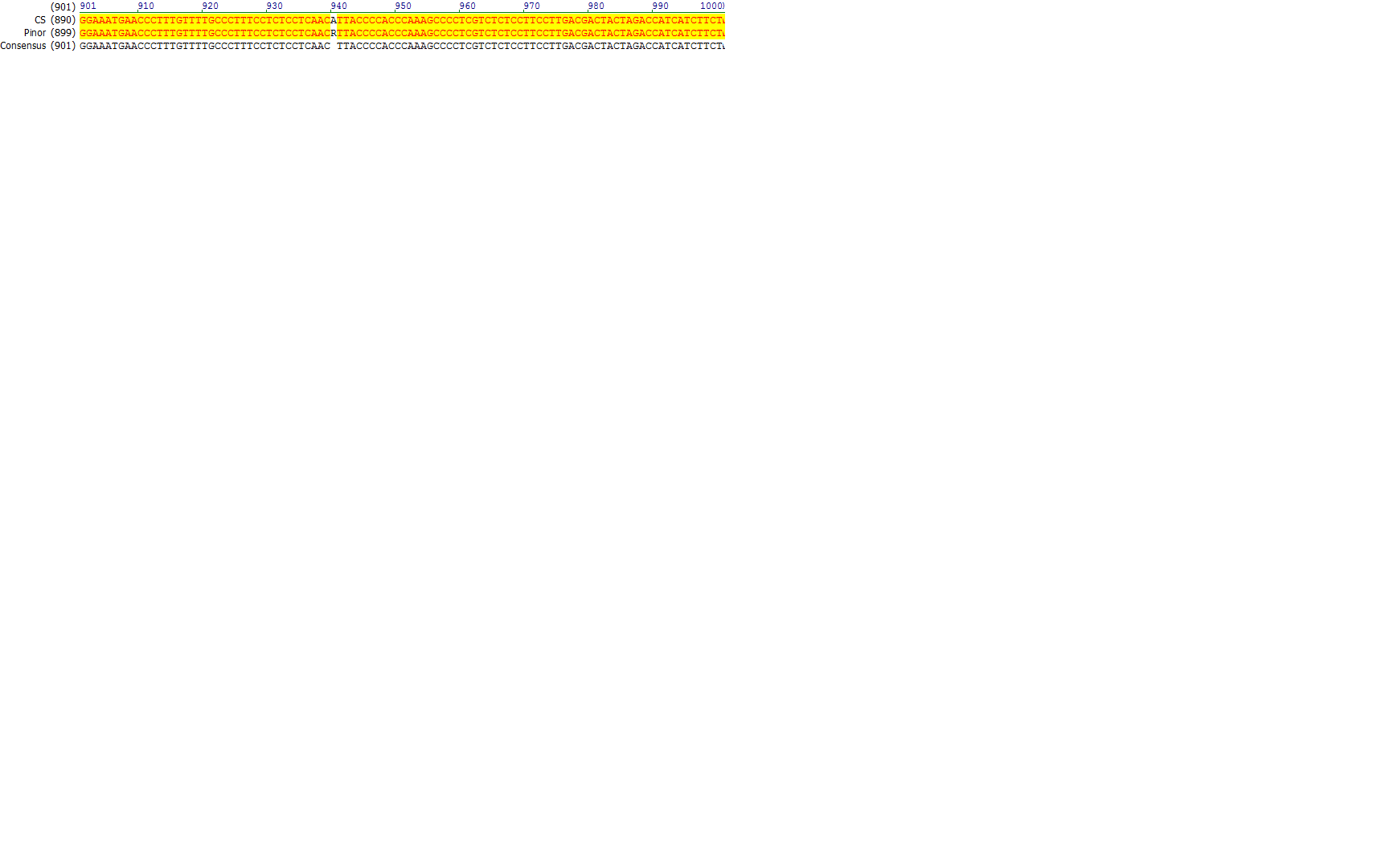

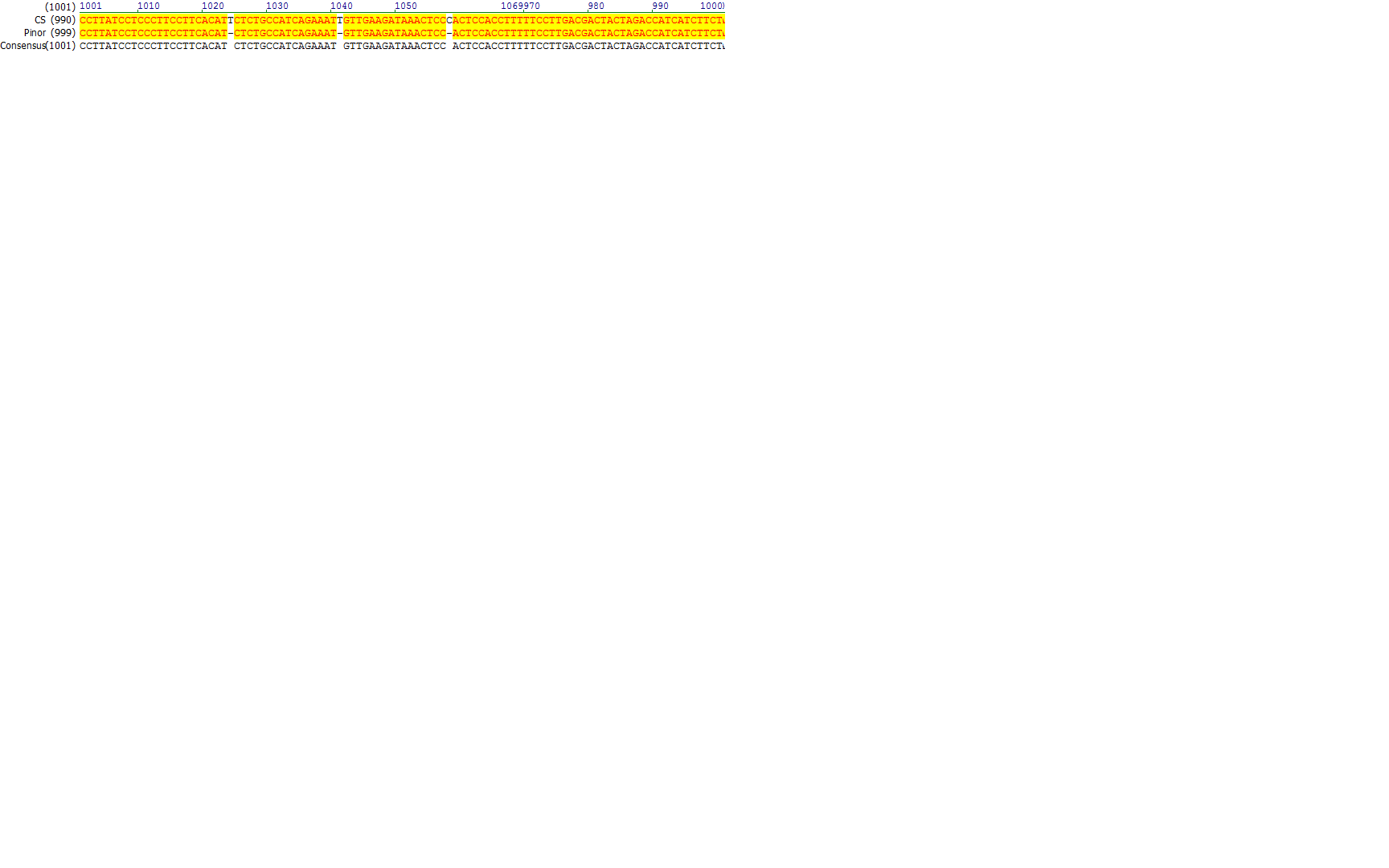


Figure S1. Alignment of the promoter sequences of *VvCCD4b* in Cabernet Sauvignon (CS) and Pinot noir (Pinot).

Table S4. *Cis*-acting elements predicted in the *VvCCD4b* promoter from Cabernet Sauvignon

| *Cis*-acting element | Sequence | Start (From TSS) | End (From TSS) | Description |
| --- | --- | --- | --- | --- |
| Box 4 | ATTAAT | -764 | -759 | Light-responsive element |
| W-box | TTGACT | -648 | -643 | Binding site for WRKY family |
| GT1 motif | GGTTAA | -549 | -544 | Light-responsive element |
| MYC binding site | CATATG | -501 | -496 | Binding site for Myc family |
| ERE | ATTTTAAA | -367 | -360 | Ethylene-responsive element |
| CCA1 binding site | AAAAATCT | -297 | -290 | Binding site for CCA1 protein |
| I box | gGATAAGGTG | -285 | -276 | Light-responsive element |
| Box-L like | ACCTTCC | -241 | -235 | Binding site for Myb family |
| CArG motif | CAAATTTAAG | -171 | -162 | Binding site for MADS family |
| CAAT | ATTTG | -97 | -93 | Binding site for CTF/NF-1 |
| TCA-element | CCATCTTTTT | -56 | -47 | Salicylic acid responsiveness |
| TATA box | TCATTATAAAA | -33 | -23 | Binding site for RNA polymerase |
| G-box | CACGTG | -22 | -17 | Light-responsive element |
| ABRE | ACGTG | -21 | -17 | Abscisic acid responsiveness |


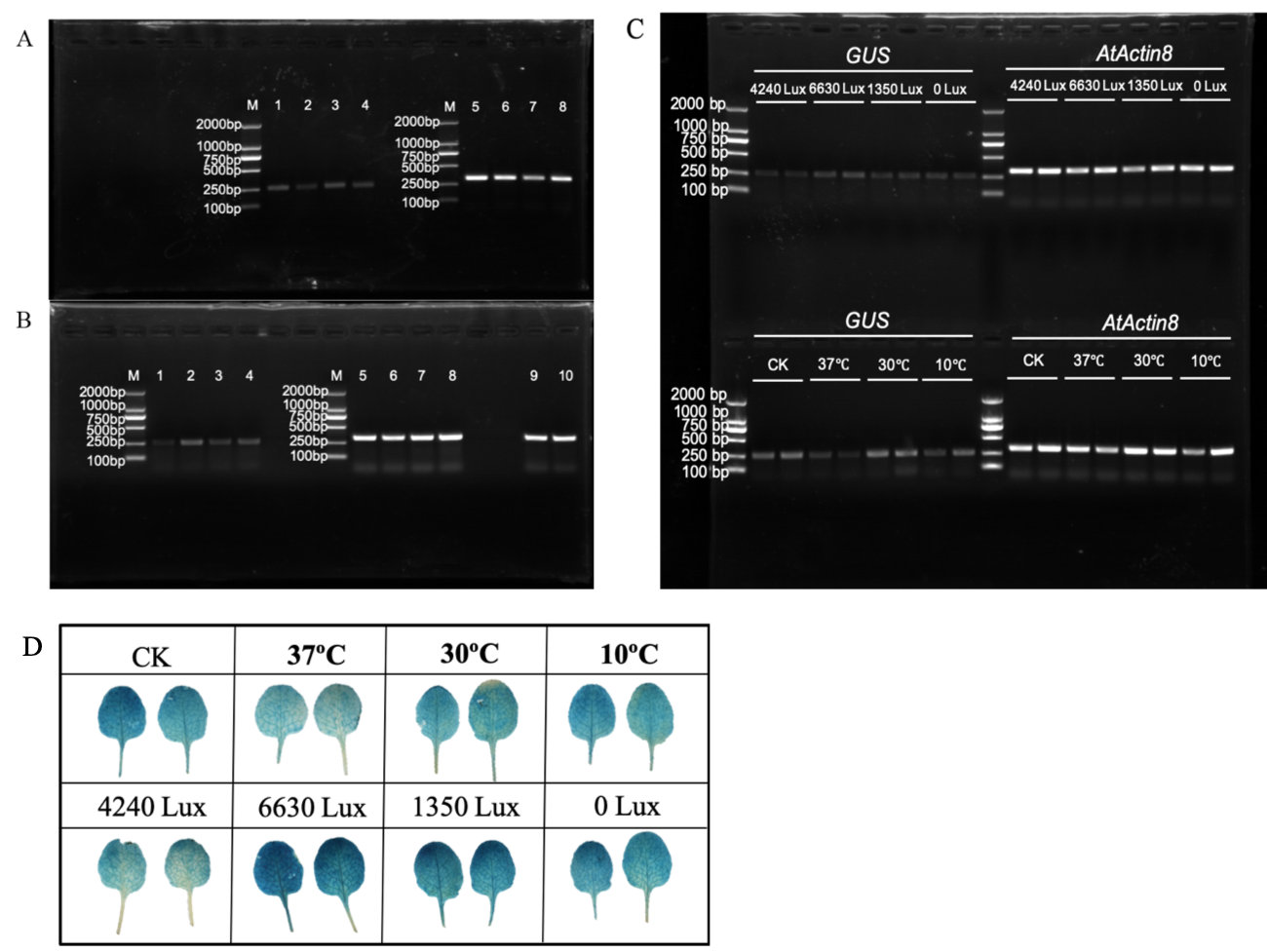


Figure S2. The original gel of *GUS* and *AtActin8* gene expression detection and GUS staining of transgenic *A. thaliana*. A: The original gel of the agarose gel in Figure 3C. M: marker DL2000. Lane 1-4: Identification of *GUS* gene expression in *VvCCD4b* promoter transgenic *A. thaliana* treated by different temperatures (CK, 37℃, 30℃ and 10℃). Lane 5-8: Identification of *AtActin8* gene expression in *VvCCD4b* promoter transgenic *A. thaliana* treated by different temperatures (CK, 37℃, 30℃ and 10℃). B. The original gel of the agarose gel in Figure 3D. M: marker DL2000. Lane 1-4: Identification of *GUS* gene expression in *VvCCD4b* promoter transgenic *A. thaliana* treated by different illuminations (4240 Lux, 6630 Lux, 1350 Lux and 0 Lux). Lane 5-8: Identification of *AtActin8* gene expression in *VvCCD4b* promoter transgenic *A. thaliana* treated by different illuminations (4240 Lux, 6630 Lux, 1350 Lux and 0 Lux). Lane 9,10: replicates of *AtActin8* gene detection in *VvCCD4b* promoter transgenic *A. thaliana* treated by light intensities. C. The agarose gel of *GUS* and *AtActin8* detection in two additional biological replicates of transgenic *A. thaliana* treated by different light or temperature. D. GUS staining of two additional biological replicates of transgenic *A. thaliana* treated by light or temperature*.*

Table S5 A list of interacting transcription factors identified by Y1H screening

| Number | Accession ID | Description |
| --- | --- | --- |
| 1 | NM_001281179.1 | *Vitis vinifera* Myb transcription factor (MYBCS1), mRNA |
| 82 | NM_001281185.1 | *Vitis vinifera* MADS-box protein 4 (MADS4), mRNA |
| 180 | XM_010654506.2 | PREDICTED: *Vitis vinifera* transcription factor MYB1R1 (LOC100253576), mRNA |
| 190 | XM_010652695.2 | PREDICTED: *Vitis vinifera* transcription factor PCL1 (LOC100253567), transcript variant X2, mRNA |
| 206 | XM_002283248.4 | PREDICTED: *Vitis vinifera* transcription factor bHLH47 (LOC100259339), transcript variant X1, mRNA |
| 221 | NM_001280990.1 | *Vitis vinifera* probable WRKY transcription factor 40-like (LOC100253480), mRNA |
| 270 | XM_002282159.4 | PREDICTED: *Vitis vinifera* bZIP transcription factor 53 (LOC100244279), mRNA |
| 304/429* | XM_010657000.2 | PREDICTED: *Vitis vinifera* GATA transcription factor 24 (LOC100251358), transcript variant X3, mRNA |
| 396 | XM_010661926.2 | PREDICTED: *Vitis vinifera* myb-related protein Myb4 (LOC100251790), mRNA |

*two numbers mean the GATA24 was screened out twice.


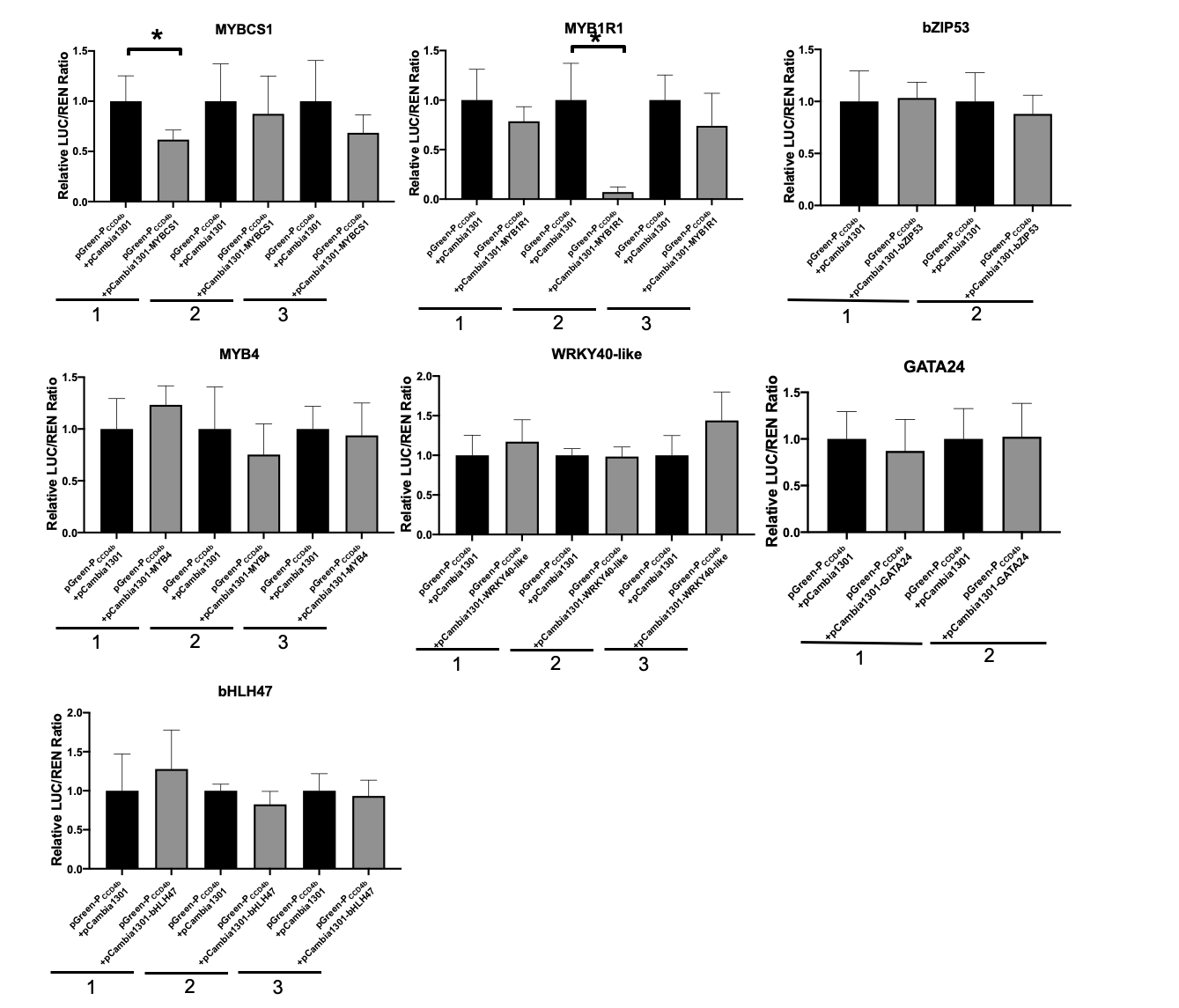


Figure S3 Regulation of transcription factors (TF) on the activity of *VvCCD4b* promoter. The dual-luciferase assay used tobacco transient expression system. pGreen-P_CCD4b_+pCAMBIA 1301 was control group, and pGreen-P_CCD4b_+pCAMBIA1301-TF was experimental group. The data were normalized by the average relative LUC/REN ratio of corresponding control group, expressed as the means ± SD from at least six biological replicates each independent experiment. The independent experiment of MYBCS1, MYB1R1, MYB4, WRKY40-like and bHLH47 was repeated for three times, respectively. The independent experiment of bHLH47 and bZIP53 was repeated for twice, respectively. The numbers below the bar chart represent repeat times of independent experiment. * indicates significant difference at 0.05.


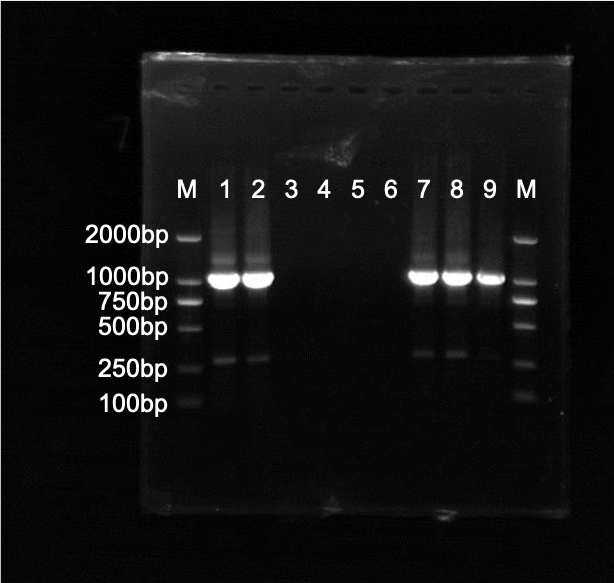


Figure S4. The original gel of hygromycin gene expression detection. M: marker DL2000. Lane 1-2: *VvMADS4* overexpression transgenic calli, Line1 and Line 2. Lane 3-6: WT. Lane 7-9: replicates of *VvMADS4* overexpression transgenic calli.


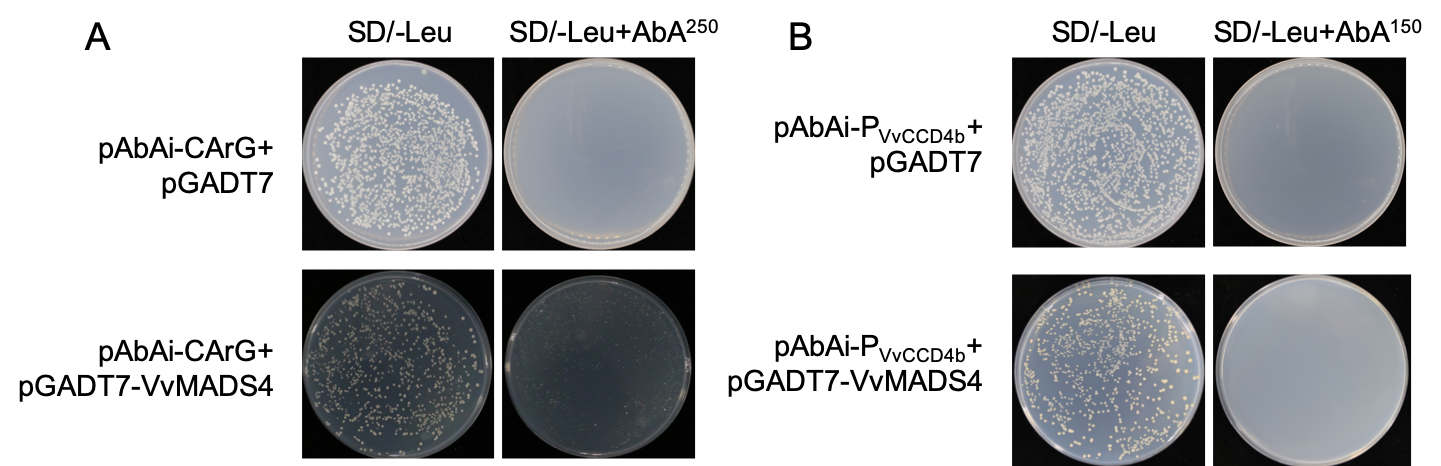


Figure S5. Interaction of the VvMADS4 protein with the *VvCCD4b* promoter.A.Y1H-hybrid assay proved the binding of VvMADS4 to the tandem CArG-box sequence. The sequence of CArG-box was the same with that on the *VvCCD4b* promoter. pAbAi-CArG+pGADT7 was control. SD/-Leu+ABA^250^ means SD/-Leu medium with 250 mg/L ABA. B. Y1H-hybrid system showing that VvMADS4 could not bind to the short fragment of *VvCCD4b* promoter which contained a CArG-box. pAbAi-P_VvCCD4b_+pGADT7 was control. SD/-Leu+ABA^150^ means SD/-Leu medium with 150 mg/L ABA.


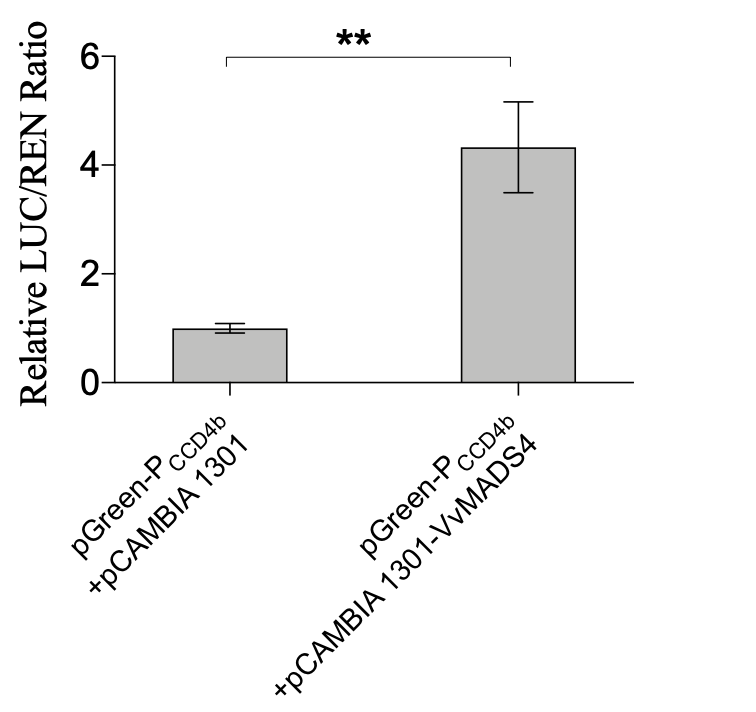


Figure S6. Transcriptional regulation of VvMADS4 on the activity of the *VvCCD4b* promoter in the *Arabidopsis* protoplast. Dual-luciferase assay showing VvMADS4 activation on the promoter activity of *VvCCD4b*. This experiment was performed using *Arabidopsis* protoplast expression system. The data are expressed as the means ± SD from at least six biological replicates. Significant difference was analyzed by AVONA. **, p<0.01.
